# The CNP analogue vosoritide mediates PDE2-sensitive anti-arrhythmogenic effects in mouse hearts with STZ-induced type 1 diabetes

**DOI:** 10.1007/s00395-025-01141-w

**Published:** 2025-09-18

**Authors:** Rebecca Firneburg, Katharina Tergau, Eleder Cachorro, Mario Schubert, Anindita Dhara, Xiaojing Luo, Erik Klapproth, Kaomei Guan, Ali El-Armouche, Susanne Kämmerer

**Affiliations:** Medical Faculty, Institute of Pharmacology and Toxicology, Technische Universität Dresden, Fetscherstraße 74, 01307 Dresden, Germany

**Keywords:** Arrhythmia, Diabetes, C-type natriuretic peptide, Vosoritide, cGMP-stimulated phosphodiesterase

## Abstract

Diabetes mellitus induces adverse structural, electrophysiological and autonomic remodelling increasing the risk for life-threatening arrhythmias, particularly after acute myocardial infarction. Natriuretic peptides (NPs) show increasing evidence of antagonising arrhythmia. Our previous study demonstrated that C-type NP (CNP) reduces arrhythmia after ischaemia–reperfusion injury (I/R) via the cGMP-dependent phosphodiesterase 2 (PDE2) in healthy mice. However, the clinical use of CNP is challenging due to its short plasma half-life. To address this, we investigated whether the more stable CNP analogue vosoritide (VO) reduces arrhythmia at cellular and organ levels in mice with STZ-induced type 1 diabetes (50 µg/g, i.p. for 5 days). After 5 weeks, STZ treatment led to elevated blood glucose and HbA1c levels, impaired cardiac function, and an increased incidence of arrhythmia after I/R in ex vivo perfused hearts. Cardiac PDE2 expression was similarly increased in diabetic mice and diabetic patients with dilated cardiomyopathy. Notably, cGMP-mediated PDE2 activation via VO clearly reduced arrhythmia generation after I/R in ex vivo perfused hearts from diabetic mice (Cohen’s d = 2.3). In cardiomyocytes, VO significantly decreased pro-arrhythmic signals upon β-adrenergic stress, such as spontaneous Ca^2+^ waves and sparks (Cohen’s d = 1.0) or L-type Ca^2+^ current amplitudes (Cohen’s d = 1.6). Specific PDE2 inhibition with BAY 60-7550 or genetic cardiomyocyte-specific PDE2 deletion prevented the anti-arrhythmic VO effects. Importantly, VO did not affect the QT interval, action potential duration, or contraction of cardiomyocytes from diabetic mice. Thus, the modified natriuretic peptide VO may serve as a promising therapeutic option to prevent lethal arrhythmias in susceptible diabetic patients.

**Supplementary Information:**

The online version contains supplementary material available at 10.1007/s00395-025-01141-w.

## Introduction

Diabetes mellitus is a well-known risk factor for cardiovascular disease, associated with increased morbidity and mortality [16]. Due to the development of coronary artery disease, diabetic patients are more likely to experience acute myocardial infarction (AMI) [34]. Cardiac ischaemia during AMI contributes to the occurrence of life-threatening arrhythmia and sudden cardiac death (SCD). The incidence of SCD is up to eightfold higher in diabetic patients compared with non-diabetic individuals, and this elevated risk appears to be independent of concomitant cardiac comorbidities [35]. Evidence suggests that amongst diabetics, people with type 1 diabetes mellitus (T1DM) are particularly at risk of SCD [21, 27]. Furthermore, diabetic autonomic neuropathy promotes arrhythmogenesis by reducing parasympathetic tone and enhancing sympathetic activity, thereby leading to higher intracellular levels of cyclic adenosine monophosphate (cAMP) and facilitating resting tachycardia, perioperative cardiovascular instability and increased mortality [17, 41]. In addition, cardiac fibrosis, systemic inflammation and electrical remodelling together create a pro-arrhythmic substrate in the heart of diabetic patients [29].

For anti-arrhythmic therapy in patients with diabetes, current recommendations for the pharmacological management of cardiac arrhythmias apply with the general principles and precautions [23]. β-Adrenoreceptor antagonists inhibiting the intracellular generation of cAMP remains the cornerstone of anti-arrhythmic pharmacotherapy, despite their potential to induce glycaemic alterations, exert negative inotropic effects and mask hypoglycaemic warning signals. Conventional ion channel blockers used in arrhythmia treatment have shown limited efficacy and may even exacerbate arrhythmogenesis through electrocardiographic changes, most notably QT prolongation [11, 32, 49]. Non-pharmacological interventions, such as implantable cardioverter-defibrillators, are highly effective in preventing SCD but do not abolish arrhythmia occurrence and carry the risks associated with surgery [4, 26]. Thus, novel therapeutic approaches are urgently needed to improve the management of arrhythmia in diabetic patients.

Natriuretic peptides (NPs) exert cardioprotective effects by binding to their transmembrane NP receptors (NPRs), which contain an intrinsic guanylate cyclase domain that generates the intracellular second messenger cyclic guanosine monophosphate (cGMP). Through this mechanism, NPs promote vasodilation and diuresis, thereby relieving the heart, and they attenuate cardiac hypertrophy and fibrosis [18]. Inhibition of NP degradation by angiotensin receptor-neprilysin inhibitors (ARNIs) enhances these beneficial actions and improves cardiac function in patients with heart failure (HF). However, the impact of ARNIs on arrhythmia development in HF remains heterogeneous across clinical studies [42]. In contrast, direct administration of C-type natriuretic peptide (CNP) was shown to reduce arrhythmia occurrence after AMI in rats [14]. We previously demonstrated that CNP diminished arrhythmic events in vivo following acute β-adrenergic stress induction as well as after ischaemia/reperfusion (I/R) in ex vivo perfused hearts from otherwise healthy mice [5]. Importantly, these effects were abolished by specific inhibition of the cGMP-dependent phosphodiesterase 2 (PDE2), providing indirect evidence that cGMP-mediated PDE2 stimulation contributes to the anti-arrhythmic CNP effects by suppressing pro-arrhythmic intracellular signalling during β-adrenergic stimulation [5].

Within the phosphodiesterase family, PDE2 has the unique property of being allosterically activated by cGMP, enhancing its cAMP-hydrolysing activity fivefold to sixfold [22]. Cardiac PDE2 expression is upregulated in human failing hearts and in animal HF models. Previous studies have shown that cardiac-specific overexpression of PDE2 reduces arrhythmogenesis following AMI in mice, whereas cardiac-specific PDE2 deletion increased the occurrence of arrhythmia following I/R in ex vivo perfused mouse hearts [45, 46].

Endogenously, CNP is released by cardiomyocytes, fibroblasts and endothelial cells. However, its clinical application is challenging due to a short plasma half-life of approximately 2 min [31]. Vosoritide (VO), a modified and longer-acting CNP analogue approved for the treatment of achondroplasia, may overcome these limitations, as it is already in clinical use [10, 37].

In this exploratory study, we investigated whether VO reduces intracellular pro-arrhythmic signals and arrhythmia in diabetic mice and human iPSC-derived cardiomyocytes (hiPSC-CMs). For this purpose, we used the extensively studied streptozotocin (STZ)-induced T1DM model in combination with ex vivo I/R injury to establish a clean diabetes model featuring both hyperglycaemia and ischaemic stress.

## Methods

A detailed and expanded methods section including IUPAC names, catalogue numbers and RRIDs of components used for the study is provided in the supplement.

### Study approval

All patients enrolled in this study gave written informed consent, and the study was conducted in accordance with the principles of the Declaration of Helsinki. Ethical approval was obtained from the institutional review committee (Official file number: EK 114082202) and the Albert Szent-Gyorgyi Medical University Ethical Review Board (Szeged). Animal experiments were performed in compliance with the ARRIVE 2.0 guidelines and in accordance with the Directive 2010/63/EU of the European Parliament on the protection of animals used for scientific purposes. All procedures were approved by the Dresden University Committee on the Use and Care of Animals (TV vG 8/2022, TVV 60/2022, TVV 49/2020).

### Human cardiac tissue samples

Human heart tissue was obtained from healthy non-failing (NF) donor hearts that were unsuitable for transplantation due to technical reasons, or from explanted hearts of end-stage NYHA III-IV DCM patients with documented insulin therapy. Patient characteristics are summarised in Supplementary Table S1. Biopsies were excised from the left ventricle.

### Mouse models

Effects of vosoritide were studied in the mouse model of streptozotocin (STZ)-induced T1DM. C57BL/6 (RRID: IMSR_RJ:C57BL-6NRJ) WT mice (8–10 weeks-old), mice with cardiomyocyte-specific deletion of PDE2 (PDE2_flox_αMHC_Cre, KO) and respective floxed control mice [5] were used in the study (Supplementary Table S2).

### Diabetes induction, blood glucose and HbA1c quantification

Diabetes was induced by i.p. injections of 50 µg/g STZ (Hycultec) for 5 consecutive days. Blood glucose levels and body weight were monitored daily during the injection phase and subsequently twice a week over a total T1DM induction period of 5 weeks. Control group animals received vehicle injections only. Venous blood glucose was determined using an Accu-Chek Aviva blood glucose system (Roche). Glycated haemoglobin A1c (HbA1c) levels were quantified in 1.6 mg/ml EDTA blood samples by ELISA using a mouse HbA1c assay kit (Crystal Chem), according to the manufacturer’s instructions.

### Echocardiography (in vivo) and ECG detection (ex vivo)

To assess cardiac performance in vivo, mice underwent echocardiography under anaesthesia (2% v/v isoflurane) using a Vevo 3100 System (VisualSonics) as described in detail in the Supplement and previously [45, 46]. To quantify arrhythmia in ex vivo perfused mouse hearts, ECG recordings were performed as described previously [40]. Briefly, following excision, hearts were cannulated and retrogradely perfused on a Langendorff system. After stabilisation, ischaemia was induced by ligation of the left anterior descending coronary artery (LAD). After 30 min, the tube was removed to allow reperfusion for an additional 30 min. QT intervals were analysed from ECGs recorded prior to occlusion.

### Cellular Ca^2+^ handling, contractility and cAMP analysis

Adult mouse ventricular myocytes were isolated by Langendorff perfusion from murine hearts following diabetes induction, as described previously [3]. Whole-cell voltage-clamp was used to measure L-type Ca^2+^ current (I_Ca,L_), as described previously [5]. Ca^2+^ spark measurements were performed in Fluo-4 AM (5 μM, Invitrogen)-loaded murine ventricular cardiomyocytes or hiPSC-CMs using the line-scan mode of a laser scanning confocal microscope (LSM 880 Pascal, Zeiss) at the Core Facility Cellular Imaging (CFCI) Dresden, Germany, as described previously [5, 20, 43]. Intracellular Ca^2+^ transients were measured at 1 Hz in Fura-2 AM-loaded (3 μM, Invitrogen) ventricular cardiomyocytes using an IonOptix system, as previously reported [2, 5]. Intracellular cAMP levels were determined in isolated cardiomyocytes using the Direct cAMP ELISA Kit (Enzo Life Sciences), and sarcomere shortening was recorded with a SarcLen Myocam system (IonOptix), as described previously [5]. Multi-electrode array (MEA) recordings were conducted according to established protocols [19].

### Differentiation and cultivation of human induced pluripotent stem cell-derived cardiomyocytes (hiPSC-CMs)

The generation of human iPSC lines was approved by the Ethics Committee of the University Medical Center Göttingen (approval numbers 21/1/11 and 10/9/15) and TU Dresden (EK422092019), and was conducted in accordance with the respective approval guidelines. hiPSC-CMs were generated from mesenchymal stem cells of a healthy donor using the STEMCCA lentiviral system and differentiated as described previously [7]. Cells were subsequently replated onto coverslips and cultured in RPMI/B27 medium for at least 1 week to recover. Thereafter, the cells were maintained in RPMI/B27 medium with normal (11 mM glucose, NG) or high (22 mM glucose, HG) concentrations of glucose for 7 days.

### Protein isolation and immunoblot

Proteins from either human or murine cardiac tissue, or from isolated murine cardiomyocytes, were isolated using RIPA buffer, separated on either 6% SDS-PAGE (ryanodine receptor) or tris-tricine gel electrophoresis and transferred onto nitrocellulose membranes. A complete list of antibodies used for protein detection is provided in the Supplement. Protein band intensities were normalised to GAPDH, whereas phosphorylated protein bands were normalised to their respective total protein. Potential diabetes-related regulation of the housekeeping protein GAPDH was assessed prior to expression analysis and showed no changes in band intensities in hearts from diabetic individuals (patients and mice) (Supplementary Data D1).

### Statistics

Animals of both sexes were randomly assigned by the supervisor of the experiments from the animal database, stratified by genotype, age and sex using an online software-based random number generator (https://www.randomizer.org/). Echocardiography and ECG detection were performed and analysed in a blinded manner. Results are presented as box plots with whiskers representing minimum to maximum values, the median and the interquartile range. The number of measurements (n/N) denotes n cells recorded from N biological replicates (animals or independent hiPSC-CM experiments).

Statistical analysis of this exploratory study was performed using GraphPad Prism software (V10; Dotmatics). Effect sizes were calculated using Cohen’s d to quantify the standardised mean difference between groups. Normality of experimental data was assessed using the D’Agostino-Pearson omnibus (K2) test. Comparisons of two groups were performed using a *t* test for normally distributed data, or Mann–Whitney test for non-normally distributed data. Welch’s correction was applied to normally distributed values with significantly different standard deviations. For comparisons of more than two groups with normally distributed data, one-way ANOVA followed by Šídák’s multiple comparisons test was used. If the Brown–Forsythe test indicated significantly different variances, Welch ANOVA followed by Dunnett’s T3 multiple comparisons test was applied. Comparisons of more than two groups with non-normally distributed data were analysed using Kruskal–Wallis test followed by Dunn’s multiple comparisons test. For cAMP levels, a RM one-way ANOVA was performed. Statistical testing of datasets with two independent variables was conducted using two-way ANOVA followed by Tukey’s test for multiple comparisons.

I_Ca,L_, Ca^2+^ imaging, single cell contractility and MEA data, as well as results from Ca^2+^ spark experiments evaluating the effects of CNP/VO were analysed using a hierarchical model in R using the lme4 package, clustering for individual mice or hiPSC-CMs batches as described previously [39]. Non-normally distributed clustered data were log-transformed before hierarchical statistical testing. Two-sided *p* values < 0.05 were considered statistically significant. As this study is exploratory, the calculated *p* values are interpreted descriptively and do not permit confirmatory statistical inference or formal hypothesis testing. Representative images were selected to closely match the average values of the respective groups.

## Results

### Diabetic patients with dilated cardiomyopathy show pro-arrhythmic cardiac remodelling

Diabetes increases the risk of developing HF and arrhythmia. To study pro-arrhythmic remodelling, we performed Western blot analysis of ventricular tissue from patients with dilated cardiomyopathy and insulin-requiring diabetes (DiabDCM), comparing them to hearts from non-diabetic patients with dilated cardiomyopathy (DCM) and to non-failing (NF) hearts.

**Fig. 1:**
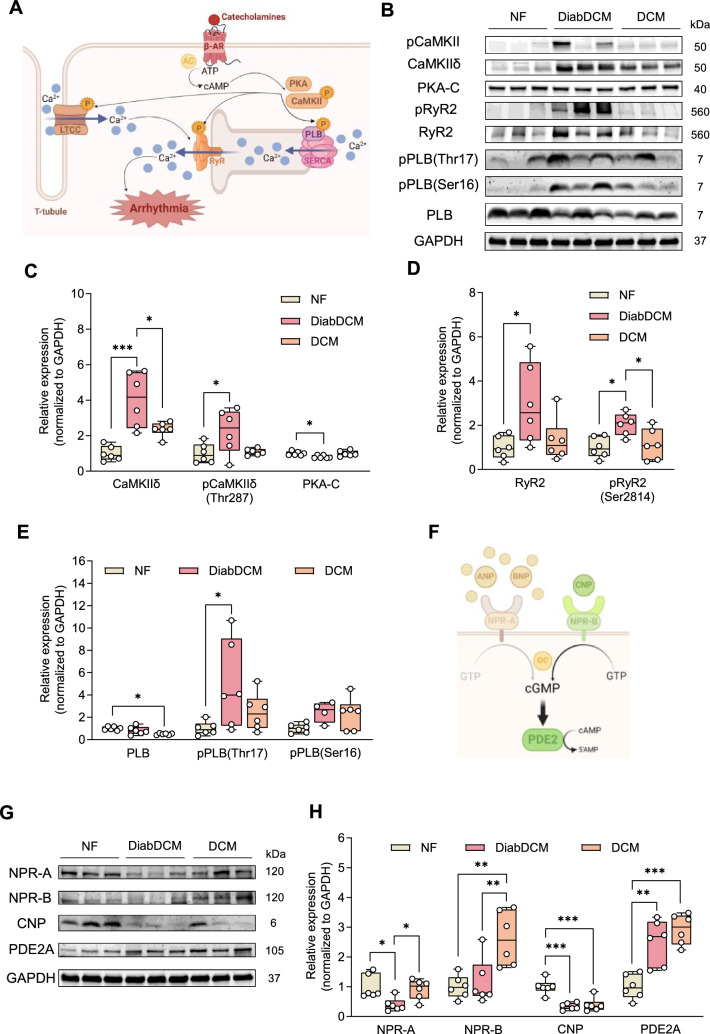
Pro-arrhythmogenic remodelling in patients with dilated cardiomyopathy with (DiabDCM) or without (DCM) insulin-requiring diabetes compared with non-failing controls (NF). **A** Illustration of proteins involved in β-adrenoreceptor (β-AR) signalling and pro-arrhythmogenic remodelling (created using BioRender.com). **B** Representative Western blots and quantification of the indicated protein expression and phosphorylation: **C** Ca^2+^/calmodulin-dependent kinase II (CaMKII) and its phosphorylation at threonine 287 (pCaMKII-Thr287), protein kinase A, catalytic subunit (PKA-C), **D** ryanodine receptor type 2 (RyR2) and its phosphorylation at serine 2814 (pRyR2-Ser2814), **E** phospholamban (PLB) and its phosphorylation at threonine 17 (pPLB-Thr17), and at serine 16 (pPLB-Ser16). **F** Schematic illustrating modulation of cAMP levels by natriuretic peptides via cGMP-dependent stimulation of phosphodiesterase 2 (PDE2) (created using BioRender.com). **G** Representative Western blots and quantification of indicated protein expression: **H** natriuretic peptide receptor A (NPR-A), natriuretic peptide receptor B (NPR-B), C-type natriuretic peptide (CNP), cGMP-dependent PDE2, *N* = 4–6 per group. Protein expressions were normalised to GAPDH, phosphorylation was normalised to total protein. Data are presented as box plots with whiskers showing minimum to maximum values, median and interquartile range. According to D’Agostino Pearson test, a normal distribution was assumed for all data.* P* values were determined by one-way ANOVA followed by Šídák’s multiple comparisons test. *p < 0.05, **p < 0.01, ***p < 0.001

Sympathetic activation in response to cardiac dysfunction elevates intracellular cAMP levels, thereby activating cardiac kinases downstream of β-adrenoreceptors (β-AR), such as Ca^2+^/calmodulin-dependent kinase II (CaMKII) and protein kinase A (PKA) (Fig. 1A). Interestingly, expression and phosphorylation of CaMKII were markedly increased in DiabDCM compared to NF or DCM heart tissue, whereas the expression of the catalytic subunit of PKA (PKA-C) was reduced (Fig. 1B, C). One key target of CaMKII- and PKA-mediated phosphorylation is the ryanodine receptor (RyR2), which mediates Ca^2+^-induced Ca^2+^ release from the sarcoplasmic reticulum (SR) during systole. Hyperactivation has been demonstrated to contribute to arrhythmogenesis due to spontaneous Ca^2+^ releases during diastole (Fig. 1A). Notably, both RyR2 expression as well as its CaMKII-dependent phosphorylation at serine 2814 (pRyR2-Ser2814) were elevated in DiabDCM hearts compared to NF or DCM tissues, suggesting an enhanced activity especially in diabetic patients (Fig. 1B, D). During diastole, phospholamban (PLB) regulates the Ca^2+^ reuptake into the SR (Fig. 1A). In comparison with NF, PLB expression was reduced in DCM, but unchanged in tissues from DiabDCM. However, its CaMKII-mediated phosphorylation at threonine 17 (pPLB-Thr17) was increased in DiabDCM, but not in Hearts from DCM. The phosphorylation level at serine 16 (pPLB-Ser16, the PKA site) remained unchanged in both (Fig. 1E). These findings indicate pronounced pro-arrhythmogenic remodelling and pathophysiological hyperphosphorylation of Ca^2+^ cycling proteins via CaMKII in DCM patients with insulin-requiring diabetes.

By activating their receptors (NPR-A and NPR-B), natriuretic peptides increase intracellular cGMP levels, thereby indirectly stimulating PDE2 (Fig. 1F). Previously, we demonstrated anti-arrhythmic effects of PDE2 in healthy mice mediated via the CNP/NPR-B/cGMP axis [5]. Consequently, we examined the expression of NPRs, CNP and PDE2 in DiabDCM tissue (Fig. 1G). Compared with NF and DCM tissues, the protein expression of the ANP/BNP receptor NPR-A was markedly downregulated in DiabDCM hearts. In contrast, the expression of the CNP-receptor NPR-B was similar in hearts from DiabDCM and NF but enhanced in non-diabetic DCM patients. Furthermore, CNP expression was decreased in both patients with and without diabetes, whereas the expression of PDE2 was clearly upregulated in DCM and DiabDCM (Fig. 1H). Taken together, these results suggest a reduced ANP/BNP signalling and a CNP deficiency in DiabDCM hearts alongside elevated PDE2 abundance.

### Failing hearts from STZ-treated mice show pro-arrhythmogenic cardiac remodelling resulting in enhanced arrhythmia susceptibility

To investigate the cellular consequences of diabetes-induced pro-arrhythmic remodelling, wild-type mice were subjected to the well-established STZ T1DM model using five consecutive daily injections of STZ (50 µg/g i.p.). After 5 weeks, STZ-treated mice (STZ) exhibited elevated plasma glucose levels and HbA1c values compared to vehicle-treated controls (Con) (Supplementary Fig. S1A, B). STZ-induced hyperglycaemia was associated with impaired cardiac function as revealed by echocardiography (Supplementary Fig. S1C). Compared with controls, STZ treatment caused a mild reduction of left ventricular ejection fraction (LVEF) and fractional area shortening (FAS) resulting in decreased cardiac outputs (CO) (Supplementary Fig. S1D). Moreover, diastolic E/e’ ratios were clearly increased, indicating diastolic dysfunction (Supplementary Fig. S1E). Consistently, cardiac expression of the stress markers, atrial natriuretic peptide (ANP) and brain natriuretic peptide (BNP), was upregulated in hearts from diabetic mice (Supplementary Fig. S1F).

**Fig. 2:**
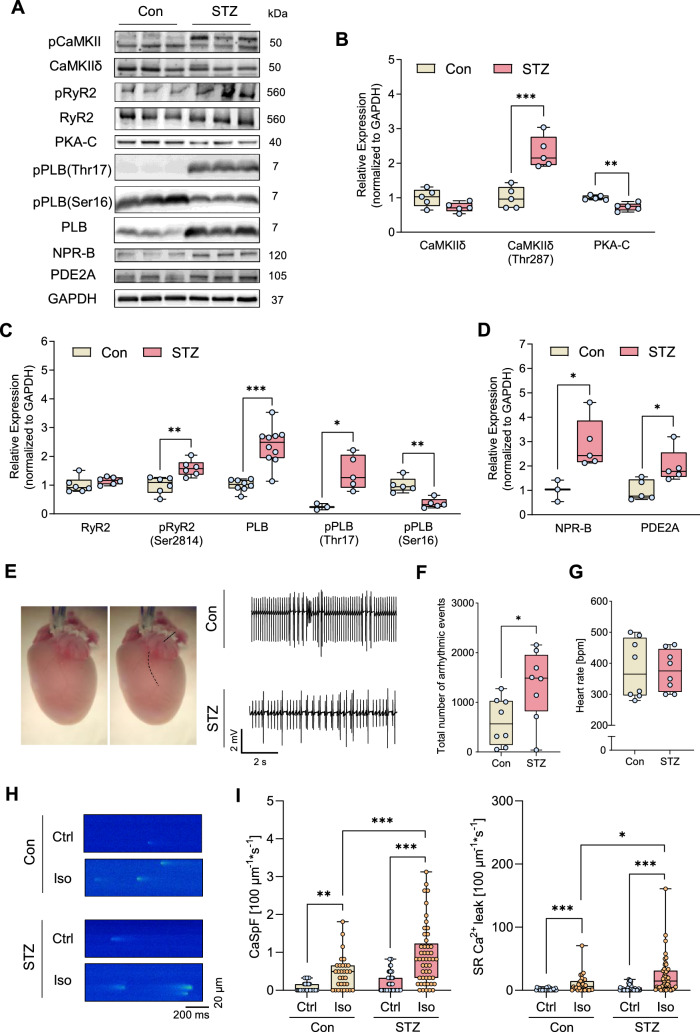
Compared with controls (Con), hearts and cardiomyocytes from mice with STZ-induced diabetes (STZ) exhibit pro-arrhythmogenic remodelling and increased arrhythmia susceptibility following ischaemia–reperfusion injury (I/R) or β-adrenergic stimulation. **A** Representative Western blots and quantification of indicated protein expression and phosphorylation in isolated ventricular cardiomyocytes from Con and STZ: **B** Ca^2+^/calmodulin-dependent kinase II (CaMKII) and its phosphorylation at threonine 287 (pCaMKII-Thr287), protein kinase A, catalytic subunit (PKA-C), **C** ryanodine receptor type 2 (RyR2) and its phosphorylation at serine 2814 (pRyR2-Ser2814), phospholamban (PLB) and its phosphorylation at threonine 17 (pPLB-Thr17), and serine 16 (pPLB-Ser16), **D** natriuretic peptide receptor B (NPR-B) and phosphodiesterase 2 (PDE2), *N*  = 3–10 per group. Protein expressions were normalised to GAPDH, phosphorylation was normalised to total protein. **E** Representative ECG traces of ex vivo perfused Con and STZ hearts after I/R. **F** Total number of arrhythmic events and **G** mean heart rates (30 min after ex vivo I/R), *N* = 8 per group. **H** Representative Ca^2+^ spark (CaSp) recordings from Con and STZ cardiomyocytes under basal conditions (Ctrl), or following stimulation with Iso (10 nM) for 7 min and pacing at 1 Hz, 10 mV for 10 s. **I** Quantification of CaSp frequency (CaSpF) and SR Ca.^2+^ leak upon the respective conditions. *n*  =  number of cells / *N* = number of animals: Con: Ctrl (30/4), Iso (31/4); STZ: Ctrl (49/7), Iso (49/7). Data are presented as box plots with whiskers showing minimum to maximum values, median and interquartile range. According to D’Agostino Pearson test, a normal distribution was assumed for **B**, **C**: pRyR2-Ser2814, PLB, pPLB-Thr17, pPLB-Ser16, **D** and **F**, whereas a non-normal distribution was assumed for **C**: RyR2, **G**, and **I**. *P* values were determined by *t* test with (**C**: PLB) or without Welch’s correction (**B**, **C**: pRyR2-Ser2814, pPLB-Thr17, pPLB-Ser16, **D**, **F**), Mann–Whitney test (**C**: RyR2, **G**) or two-way ANOVA (**I**). *p < 0.05, **p < 0.01, ***p < 0.001

To validate the comparability of the STZ model with the human heart, the molecular composition of the proteins involved in the β-AR signalling pathway was investigated in ventricular cardiomyocytes from STZ-treated mice and compared to those from healthy controls (Con). Similar to the findings in DCM patients with diabetes, the expression and, notably, the phosphorylation levels of cAMP-related kinases and Ca^2+^-regulating proteins were altered in cardiomyocytes from diabetic mice (Fig. 2A), potentially contributing to impaired cardiac performance. Compared to Con, cardiomyocytes from diabetic mice displayed a reduced expression of PKA-C, indicating a reduced PKA activity. In contrast, CaMKII expression was unchanged. However, its phosphorylation at threonine 287 (pCaMKII-Thr287) was clearly higher, indicating increased activity (Fig. 2B). Whilst RyR2 expression levels were unaltered in cardiomyocytes from STZ-treated mice, this led to enhanced phosphorylation of RyR2 at the CaMKII site Ser2814 and was further reflected by elevated phosphorylation of PLB at the CaMKII site threonine 17 (pPLB-Thr17). In contrast, the PKA-dependent phosphorylation at serine 16 (pPLB-Ser16) was markedly reduced (Fig. 2C). Taken together, hearts from STZ-treated animals, like those of DiabDCM patients, exhibit altered protein expression within the β-adrenergic cAMP signalling pathway, characterised by an enhanced role of CaMKII and CaMKII-dependent phosphorylation.

Diabetes is associated with an increased risk of AMI, ventricular arrhythmia and ultimately SCD [1]. To assess arrhythmia susceptibility in hearts from diabetic mice, the occurrence of arrhythmia was quantified following ischaemia induction by transient ligation of the left anterior descending artery (LAD) for 30 min followed by reperfusion (I/R) in ex vivo perfused diabetic hearts and compared to non-diabetic control hearts. Hearts from STZ-treated wild-type mice exhibited higher numbers of arrhythmic events after I/R than those from non-diabetic vehicle-treated controls (Con) (Fig. 2E). Notably, the overall number of arrhythmic events was markedly increased (Fig. 2F), whereas heart rates remained unchanged (Fig. 2G). The incidence of ventricular extrasystoles (VES), bigeminy and couplets also tended to be increased in STZ hearts (Supplementary Fig. S1G). At the cellular level, isolated cardiomyocytes from STZ-treated animals displayed a greater number of spontaneous Ca^2+^ releases (Ca^2+^ sparks, CaSp) from the SR in response to β-adrenergic stimulation with isoprenaline (Iso, 10 nM) compared to cells from healthy controls. Furthermore, this led to an increased total SR Ca^2+^ leak, reflecting CaSp amplitude, duration, width, and frequency, in cells from diabetic mice (Fig. 2H,I). Interestingly, the increased arrhythmia susceptibility observed in hearts and cardiomyocytes from diabetic mice was accompanied by elevated expression of NPR-B and PDE2 (Fig. 2D).

### Pharmacological PDE2 activation by CNP and its analogue vosoritide reduces isoprenaline-induced pro-arrhythmogenic Ca^2+^ sparks in cardiomyocytes from diabetic mice

Previously, we demonstrated that cGMP-dependent activation of PDE2 via CNP effectively suppresses pro-arrhythmic Ca^2+^ signals induced by β-adrenergic stimulation in cardiomyocytes from healthy mice [5]. We, therefore, investigated whether PDE2 stimulation also exerts anti-arrhythmic effects under diabetic conditions in cardiomyocytes from STZ-treated mice. Following the same protocol as previously published, we quantified spontaneous CaSp in isolated cardiomyocytes from diabetic wild-type (WT) mice (Supplementary Fig. 2A). Like in our experiments conducted in cardiomyocytes from healthy mice, β-adrenergic stimulation with Iso (10 nM) increased CaSp frequency (CaSpF) as well as the total SR Ca^2+^ leak. Importantly, CNP (1 µM) markedly reduced the Iso-induced CaSpF as well as the total Ca^2+^ leak, whereas concomitant PDE2 inhibition with the specific PDE2 inhibitor BAY 60–7550 (BAY, 100 nM) abolished the protective effects of CNP in cells from diabetic WT mice (Supplementary Fig. 2B). Moreover, the anti-arrhythmic effects of CNP were blunted in cardiomyocytes with cardiac-specific PDE2 knockout (KO) (Supplementary Fig. 2C, D). These findings indicate that, even under STZ-induced diabetic remodelling, CNP-dependent PDE2 activation mitigates pro-arrhythmic Ca^2+^ release.

**Fig. 3:**
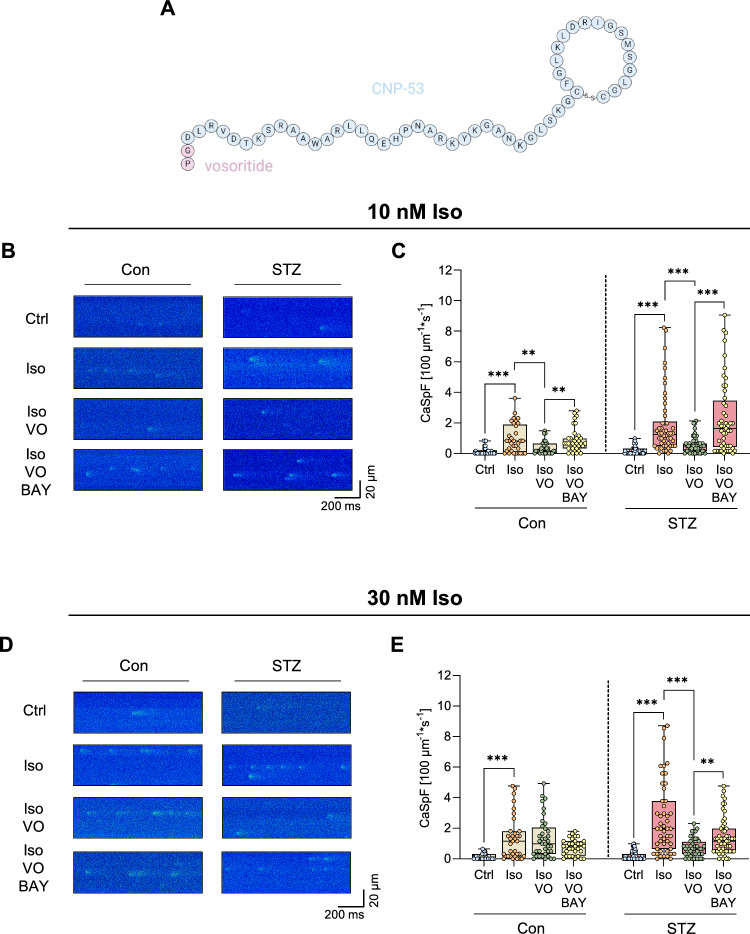
cGMP-dependent PDE2 stimulation with vosoritide (VO) protects ventricular cardiomyocytes isolated from diabetic mice against pro-arrhythmogenic intracellular Ca.^2+^ sparks (CaSp). **A** Schematic structure showing the CNP analogue vosoritide (VO) and the bioactive CNP fragment CNP-53, created with *Marvin* for JavaScript. **B** Representative CaSp recordings in cells from wild-type mice without (Con) or with STZ-induced diabetes (STZ) under basal conditions (Ctrl), or following stimulation with Iso (10 nM), Iso + VO (1 µM), or Iso + VO + BAY 60–7550 (BAY, 100 nM) for 7 min and pacing at 1 Hz, 10 mV for 10 s. **C** Quantification of CaSp frequency (CaSpF) under the respective conditions, *n* = number of cells / *N* = number of animals: Con: Ctrl (30/4), Iso (33/4), Iso + VO (31/4), Iso + VO + BAY (35/4); STZ: Ctrl (50/7), Iso (50/7), Iso + VO (52/7), Iso + VO + BAY (49/7). **D** Representative CaSp recordings in cells from control animals (Con) and mice with STZ-induced diabetes (STZ) under basal conditions (Ctrl), or following stimulation with Iso (30 nM), Iso + VO (1 µM), or Iso + VO + BAY (100 nM) for 7 min and pacing at 1 Hz, 10 mV for 10 s. **E** Quantification of CaSpF under the respective conditions, *n* = number of cells / *N* = number of animals: Con: Ctrl (34/4), Iso (35/4), Iso + VO (37/4), Iso + VO + BAY (32/4); STZ: Ctrl (51/7), Iso (49/7), Iso + VO (48/7), Iso + VO + BAY (55/7). Data are presented as box plots with whiskers showing minimum to maximum values, median and interquartile range. According to D’Agostino Pearson test, all data were assumed to be non-normally distributed. *p* values were determined by Bonferroni test after a hierarchical model. *p < 0.05, **p < 0.01, ***p < 0.001

However, as outlined above, the clinical application of CNP is limited by its short half-life. To overcome this, modified NPs were developed. The CNP analogue vosoritide (VO), which is approved for the therapy of achondroplasia, consists of the bioactive fragment CNP-53 with two additional Amino acids at the N-terminus, extending the plasma half-life to approximately 30 min (Fig. 3A) [6]. Given the anti-arrhythmogenic effects of CNP, we next investigated whether VO similarly reduces pro-arrhythmogenic CaSp. Indeed, under concomitant stimulation with Iso (10 nM), VO (1 µM) considerably suppressed the Iso-induced increase in CaSpF in cardiomyocytes from both control (Con) and STZ-treated (STZ) mice. Importantly, this effect was abolished by PDE2 inhibition with BAY (100 nM), indicating that VO exerts anti-arrhythmic effects via PDE2 activation (Fig. 3B, C). Similarly, VO reduced the total Iso-induced Ca^2+^ leak in cardiomyocytes from diabetic WT mice, whereas BAY co-treatment enhanced it (Supplementary Fig. S3A).

Cardiac dysfunction is associated with sympathetic overactivity, which enhances β-adrenergic stress in the heart and thereby promotes arrhythmogenesis [13]. To mimic this condition, we increased the Iso concentration to 30 nM in our experimental protocol and tested whether the anti-arrhythmogenic effect of VO (1 µM) was preserved under higher β-adrenergic stimulation. Interestingly, whilst the protective effect of VO was blunted in Con cardiomyocytes co-stimulated with Iso (30 nM), VO still mediated a relevant anti-arrhythmogenic effect in cardiomyocytes from STZ-treated mice (Cohen’s d = 1.096) (Fig. 3D, E, Supplementary Fig. S3B). Notably, this effect was abolished by co-incubation with BAY (100 nM) suggesting that the upregulation of PDE2 in hearts from STZ-treated mice amplifies the protective action of VO.

### Vosoritide protects cardiomyocytes from diabetic mice against β-adrenergic stimulation-induced arrhythmogenic Ca^2+^ influxes and Ca^2+^ waves

**Fig. 4:**
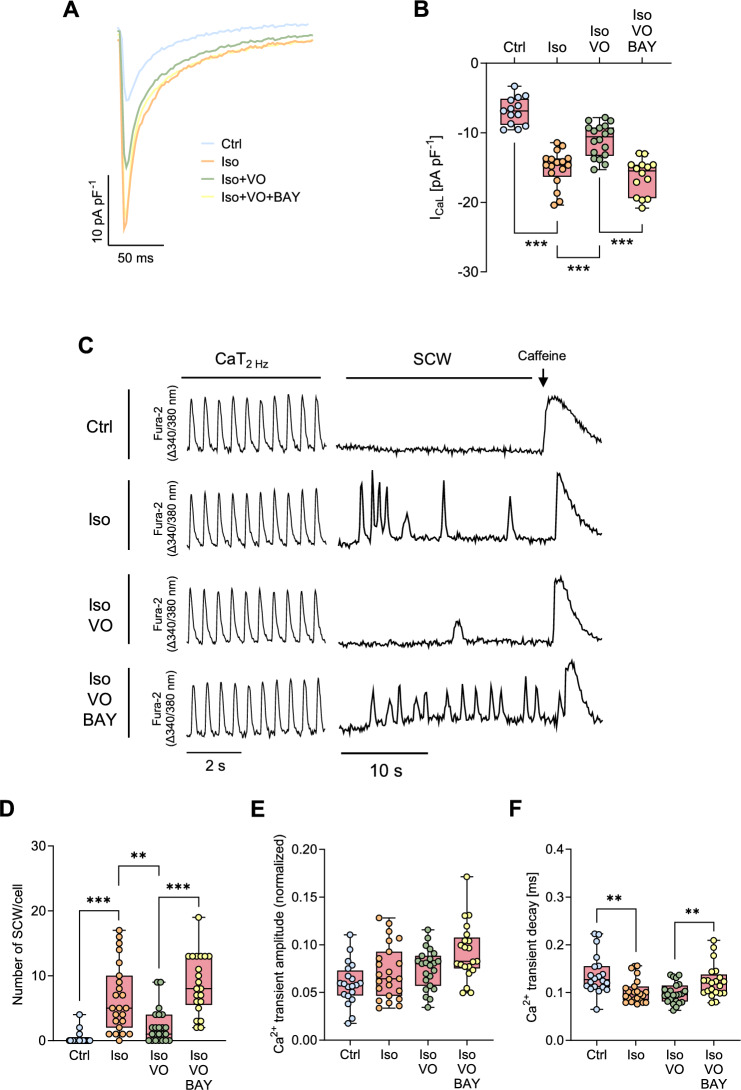
Vosoritide (VO) reduces pro-arrhythmogenic Ca^2+^ influx and spontaneous Ca^2+^ waves via PDE2 in ventricular cardiomyocytes isolated from diabetic mice. **A** Representative I_Ca,L_ recordings in cells from diabetic wild-type mice under basal conditions (Ctrl), or following stimulation with Iso (30 nM), Iso + VO (10 µM), or Iso + VO + BAY 60–7550 (BAY, 300 nM) for 10 min. **B** I_Ca,L_ current density measured at 0 mV under the respective conditions, *n* = number of cells / *N* = number of animals: Ctrl (13/10), Iso (17/10), Iso + VO (18/9), Iso + VO + BAY (14/7). **C** Representative traces of ventricular cardiomyocytes from diabetic wild-type mice loaded with Fura-2, under basal conditions (Ctrl), or following stimulation with Iso (30 nM), Iso + VO (10 μM), or Iso + VO + BAY (300 nM) for 10 min and subjected to arrhythmia provocation (pacing at 2 Hz, 10 mV for 30 s). **D** Quantification of spontaneous calcium waves (SCW) per cell, **E** Ca^2+^ transient amplitude at 2 Hz and **F** Ca.^2+^ transient decay at 2 Hz under the respective conditions, *n* = number of cells / *N* = number of animals: Ctrl (20/6), Iso (23/6), Iso + VO (23/6), Iso + VO + BAY (21/6). Data are presented as box plots with whiskers showing minimum to maximum values, median, and interquartile range. According to D’Agostino Pearson test, a normal distribution was assumed for **B** and **E**, whereas a non-normal distribution was assumed for **D** and **F**. *p* values were determined by Bonferroni test after a hierarchical model. *p < 0.05, **p < 0.01, ***p < 0.001

Ca^2+^ influx via L-type Ca^2+^ channels triggers a Ca^2+^-mediated Ca^2+^ release from the SR, thereby contributing to arrhythmogenesis [28]. To further study the potential anti-arrhythmic effects of VO, we analysed the effects of VO-mediated PDE2 activation on L-type Ca^2+^ current (I_Ca,L_) density under β-adrenergic stimulation. Importantly, the Iso-induced increase in peak I_Ca,L_ density at 0 mV was clearly diminished by VO (Cohen’s d = 1.620), whereas co-application of the PDE2 inhibitor BAY reversed this effect, restoring Ca^2+^ influx in WT cardiomyocytes from diabetic mice (Fig. 4A, B).

So far, we demonstrated that VO suppresses small Iso-induced Ca^2+^ releases via both L-type Ca^2+^ channels at the plasma membrane and RyR channels in the SR through PDE2 activation. However, the enhanced I_Ca,L_ as well as multiple CaSp can trigger large SR Ca^2+^ releases, resulting in arrhythmogenic Ca^2+^ waves and contraction. To test whether VO protects against such events, we assessed intracellular Ca^2+^ transients and the occurrence of spontaneous Ca^2+^ waves (SCW) after pacing at 2 Hz in cardiomyocytes from diabetic mice (Fig. 4C). Iso markedly increased the number of SCW in cardiomyocytes from diabetic animals, whereas VO substantially reduced Iso-induced SCW (Cohen’s d = 1.047), indicating a suppression of pro-arrhythmic contractions. This effect was abolished by simultaneous PDE2 inhibition with BAY (Fig. 4D). In cardiomyocytes from diabetic mice, the Ca^2+^ transient amplitude was not statistically significantly affected by Iso, VO or BAY (Fig. 4E). In contrast, Iso accelerated SR Ca^2+^ reuptake kinetics, whilst concomitant VO stimulation had no additional effect. PDE2 inhibition with BAY slightly slowed the Iso-induced time constant τ of the Ca^2+^ decay (Fig. 4F). Together, these results demonstrate that VO prevents arrhythmogenic Ca^2+^ waves in cardiomyocytes from diabetic mice, without impairing rhythmic Ca^2+^ cycling during β-adrenergic stimulation.

### Genetic PDE2 deletion prevents cellular anti-arrhythmogenic effects of vosoritide

To validate that the anti-arrhythmogenic effects of VO are mediated via PDE2, pro-arrhythmic Ca^2+^ releases and currents in isolated cardiomyocytes were quantified in diabetic mice with cardiac-specific PDE2 deletion (PDE2 KO) subjected to STZ treatment. Consistent with the effects of pharmacological PDE2 inhibition, VO did not affect the Iso-induced increase in CaSpF or the SR Ca^2+^ leak in cells from diabetic PDE2 KO animals (Supplementary Fig. S4A, B). Similarly, patch-clamp experiments revealed that genetic PDE2 deletion prevented the VO-mediated reduction of I_Ca,L_, displaying comparable current densities at 0 mV under stimulation with both, Iso alone or Iso plus VO (Supplementary Fig. S4C, D). VO also failed to decrease Iso-mediated pro-arrhythmogenic SCW in PDE2-deficient cardiomyocytes, whilst Ca^2+^ transient amplitudes remained unaffected, as in WT cells (Supplementary Fig. S4E). Together, these results indicate that VO protects against pro-arrhythmic Ca^2+^ signals in cardiomyocytes from diabetic mice via cGMP-dependent PDE2 activation, an effect lost upon genetic deletion or pharmacological inhibition of PDE2. This supports the critical role of the NPR-B/cGMP/PDE2/cAMP axis in limiting pro-arrhythmogenic Ca^2+^ releases in cardiomyocytes.

### Vosoritide prevents arrhythmia generation ex vivo after ischaemia–reperfusion injury

**Fig. 5:**
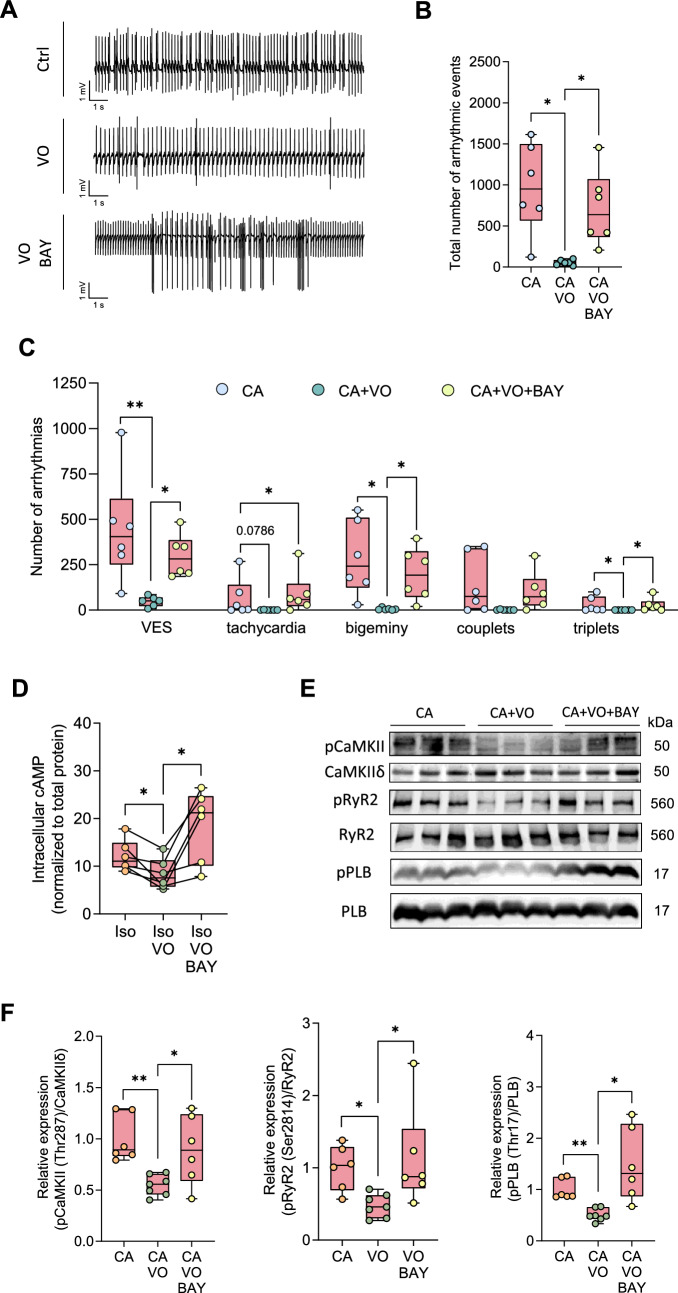
Vosoritide (VO) protects hearts from diabetic mice against arrhythmia following ex vivo ischaemia/reperfusion injury (I/R) by stimulating PDE2 and reducing cAMP/CaMKII-dependent phosphorylation of Ca^2+^ cycling proteins at the cellular level.** A** Representative ECG traces, **B** total number of arrhythmic events, **C** number of ventricular extrasystoles (VES), tachycardia, bigeminy, couplets and triplets, in hearts from STZ-treated mice perfused ex vivo with Krebs–Henseleit buffer containing physiological catecholamine (CA) concentrations (10 nM norepinephrine, 3.5 nM epinephrine) with or without vosoritide (200 nm), or BAY 60-7550 (BAY, 300 nm) after I/R, *N*  = 6 per group. **D** cAMP content in isolated ventricular cardiomyocytes quantified by direct cAMP ELISA following treatment with Iso (10 nM), Iso + VO (1 µM), or Iso + VO + BAY (100 nM) for 10 min, *N*  = 6 per group. Relative cAMP concentrations were normalised to corresponding sample protein content and the Iso group. **E** Representative Western blots, and **F** quantification of Ca^2+^/calmodulin-dependent kinase II (CaMKII) phosphorylation at threonine 287 (pCaMKII-Thr287) and CaMKII-dependent phosphorylation of ryanodine receptor 2 at serine 2814 (pRyR2-Ser2814) and phospholamban at threonine 17 (pPLB-Thr17) in murine ventricular heart tissue following ex vivo Langendorff perfusion with Krebs–Henseleit buffer containing catecholamines (CA, 10 nM norepinephrine, 3.5 nM epinephrine), CA + VO (200 nM), or CA + VO + BAY (300 nM) for 1:15 h, *N* = 6 (CA, CA + VO + BAY), or *N* = 7 (CA + VO) per group. Protein phosphorylation was normalised to total protein. Data are presented as box plots with whiskers showing minimum to maximum values, median, and interquartile range. According to D’Agostino Pearson test, normal distribution was assumed for **B**, **C**: bigeminy, couplets, **D**, **F**: pCaMKII-Thr287, pPLB-Thr17, non-normal distribution was assumed for **C**: VES, tachycardia, triplets and **F**: pRyR2-Ser2814. *p* values were determined by Dunnett’s T3 multiple comparisons test after Brown–Forsythe test and Welch ANOVA (**B**, **C**: bigeminy, F: pPLB-Thr17), Šídák’s multiple comparisons test after one-way ANOVA (**C**: couplets, **F**: pCaMKII-Thr287), RM one-way ANOVA (**D**), or Dunn’s multiple comparisons test after Kruskal–Wallis test (**C**: VES, tachycardia, triplets, **F**: pRyR2-Ser2814). *p < 0.05, **p < 0.01

As a proof of concept for the potential anti-arrhythmic effect of VO, we perfused isolated hearts from diabetic WT mice and quantified arrhythmic events after I/R (Fig. 5A). In diabetic hearts, VO markedly reduced the occurrence of arrhythmic events (Cohen’s d = 2.340), whereas co-perfusion with the PDE2 inhibitor BAY abolished this protective effect (Fig. 5B). When differentiating amongst arrhythmia types, VO decreased the number of VES, bigeminy, triplets and couplets, whilst BAY increased their occurrence (Fig. 5C). These results highlight the anti-arrhythmic potential of vosoritide after AMI in hearts of diabetic individuals.

### Vosoritide reduces catecholamine-induced cellular cAMP levels and phosphorylation of Ca^2+^ handling proteins

To validate the proposed mechanism that VO reduces β-AR/cAMP-mediated arrhythmia development in diabetic cardiomyopathy via PDE2 activation, intracellular cAMP levels were measured in isolated cardiomyocytes from STZ-treated mice. ELISA assay revealed that VO clearly attenuated Iso-induced intracellular cAMP accumulation, whereas simultaneous PDE2 inhibition restored cAMP levels (Fig. 5D). The downstream effects of VO-mediated intracellular cAMP reduction on phosphorylation of CaMKII at threonine 287 (pCaMKII-Thr287), as well as its targets RyR2 at serine 2814 (pRyR2-Ser2814) and PLB at threonine 17 (pPLB-Thr17), which contribute to arrhythmogenesis, were quantified (Fig. 5E). Normalised to total protein expression levels, VO reduced CaMKII phosphorylation, indicating decreased kinase activity, whilst PDE2 inhibition reversed this effect. Consistently, Iso-induced phosphorylation of RyR2 and PLB at their CaMKII sites was diminished by VO, likely mediating reduced pro-arrhythmogenic SR Ca^2+^ releases. This effect was prevented by PDE2 inhibition, which restored RyR2 and PLB phosphorylation levels (Fig. 5F). These results provide mechanistic evidence that the anti-arrhythmic effects of VO are mediated by reduced intracellular cAMP levels and consequent suppression of CaMKII-dependent phosphorylation of Ca^2+^ handling proteins.

### Vosoritide does not affect cardiac action potentials and cellular contraction

**Fig. 6:**
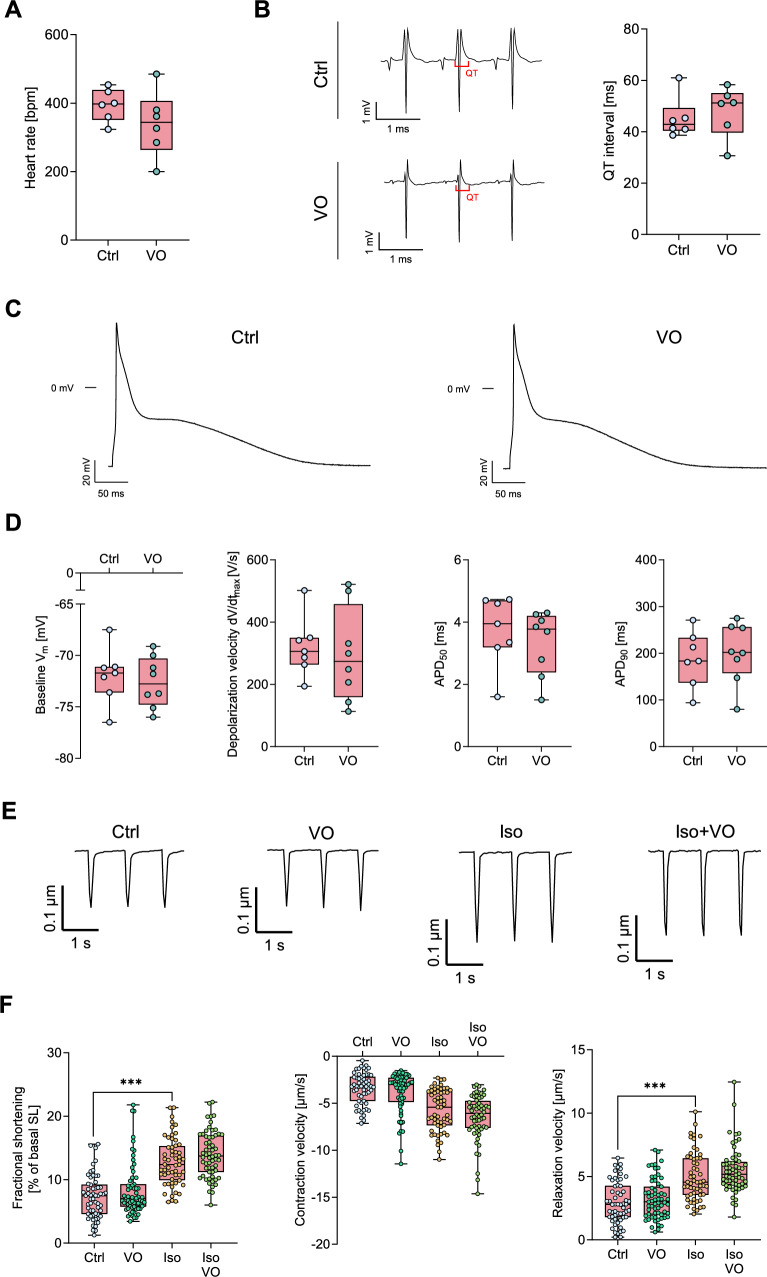
Vosoritide (VO) does not affect heart rate or QT interval in ex vivo perfused hearts, nor action potential and contraction properties of ventricular cardiomyocytes isolated from diabetic mice. **A** Heart rate in diabetic hearts perfused ex vivo with Krebs–Henseleit buffer containing physiological catecholamine concentrations (10 nM norepinephrine, 3.5 nM epinephrine) without (Ctrl) or with VO (200 nM), *N* = 6 per group. **B** Representative ECG recordings and quantification of QT intervals of diabetic hearts perfused ex vivo with Krebs–Henseleit buffer containing physiological catecholamine concentrations (10 nM norepinephrine, 3.5 nM epinephrine) without (Ctrl) or with VO (200 nM), *N* = 6 per group. **C** Representative action potential recordings in cardiomyocytes kept in bath solution with or without VO (10 µM). **D** Baseline membrane potential, depolarisation velocity, and action potential duration at 50% (APD_50_) and 90% (APD_90_) repolarisation, *n* = number of cells / *N* = number of animals: Ctrl (7/4), VO (8/4). **E** Representative traces of sarcomere shortening under control conditions (Ctrl) and following stimulation with VO (1 µM), Iso (10 nM), or Iso + VO for 5 min and pacing at 1 Hz, 10 mV. **F** Sarcomere shortening amplitude normalised to baseline sarcomere length, contraction velocity and relaxation velocity in cardiomyocytes under the respective conditions, *n* = number of cells / *N* = number of animals: Ctrl (58/6), VO (63/6), Iso (56/6), Iso + VO (60/6). Data are presented as box plots with whiskers showing minimum to maximum values, median and interquartile range. According to D’Agostino Pearson test, a normal distribution was assumed for **A**, **B**, **D,** whereas a non-normal distribution was assumed for **F**. *p* values were determined by *t* test (**A**, **B**, **D**) or by Bonferroni test after a hierarchical model (**F**). ***p < 0.001

Next, we assessed the impact of VO on electrophysiological and contractile properties of cardiomyocytes isolated from diabetic mice. In ex vivo perfused diabetic hearts, VO did not alter QT interval or heart rate compared to controls. (Fig. 6A, B). At the cellular level, action potentials (AP) were recorded in isolated cardiomyocytes from diabetic WT mice in the absence and presence of VO (Fig. 6C). VO did not affect key AP parameters, including resting membrane potential, maximal upstroke velocity, or AP duration at 50 and 90% repolarisation (Fig. 6D).

To evaluate contractile function, fractional shortening and contraction/relaxation velocities were measured in cardiomyocytes from STZ-treated WT under Iso, VO, or PDE2 inhibition (Fig. 6E). As expected, Iso enhanced the fractional cell shortening compared to controls. In contrast, VO did not affect basal fractional shortening or basal contraction and relaxation velocities, nor did it affect the Iso-induced enhancement of contraction parameters in cardiomyocytes from diabetic mice (Fig. 6F). These results indicate that VO exerts its anti-arrhythmic effects without altering baseline electrophysiology or contractile function in cardiomyocytes from diabetic animals.

### The anti-arrhythmogenic effects of vosoritide persist in human iPSC-derived cardiomyocytes

**Fig. 7:**
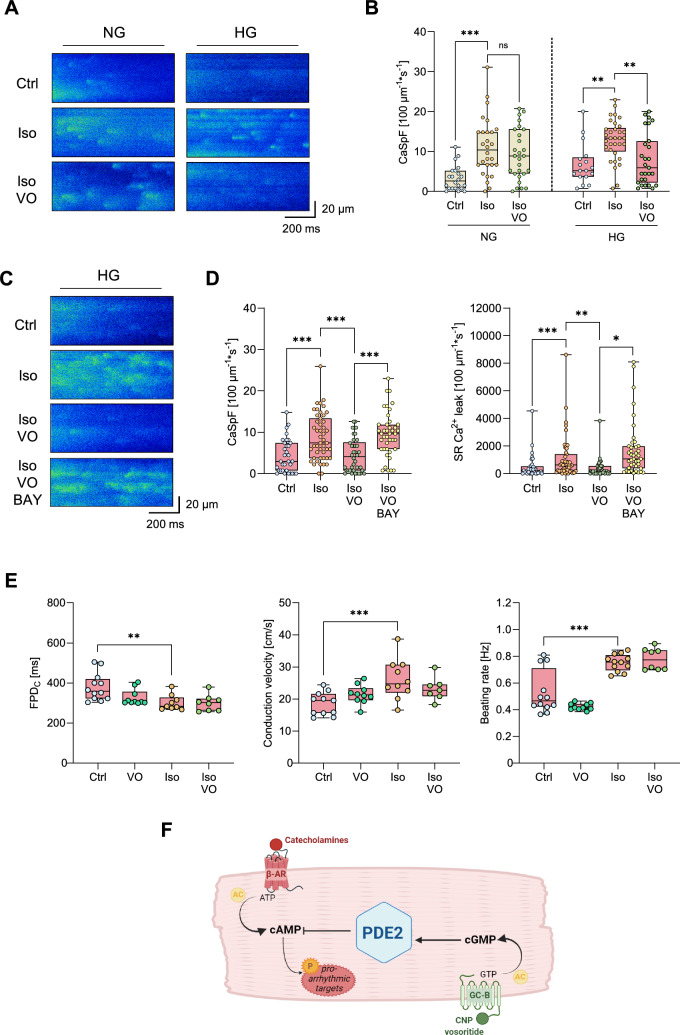
Vosoritide (VO) reduces Ca^2+^ sparks (CaSp) in human iPSC-derived cardiomyocytes (hiPSC-CMs) cultured under high glucose (HG) conditions via activation of PDE2, without affecting cellular electrophysiological parameters. **A** Representative CaSp recordings of hiPSC-CMs cultured under normo (NG) or high (HG) glucose conditions under basal conditions (Ctrl), or following stimulation with Iso (100 nM), or Iso + VO (1 µM) for 5 min and pacing at 0.5 Hz, 10 mV. **B** Quantification of CaSp frequency under the respective conditions (CaSpF), *n* = number of cells / *N* = number of independent experiments: NG: Ctrl (22/4), Iso (28/4), Iso + VO (27/4); HG: Ctrl (17/3), Iso (33/4), Iso + VO (27/4). **C** Representative CaSp recordings of HG-hiPSC-CMs under basal conditions (Ctrl), or following stimulation with Iso (100 nM), Iso + VO (1 µM), or Iso + VO + BAY 60–7550 (BAY, 100 nM) for 5 min and pacing at 0.5 Hz, 10 mV. **D** Quantification of CaSpF and SR Ca.^2+^ leak under the respective conditions, *n* = number of cells / *N* = number of independent experiments: Ctrl (33/4), Iso (47/4), Iso + VO (38/4), Iso + VO + BAY (41/4). **E** Effects of VO (1 µM), Iso (100 nM) or Iso + VO on field potential duration corrected for beating frequency using Fridericia’s formula (FPD_C_,), conduction velocity and beating rate of HG-hiPSC-CMs. *n* = number of cells / *N* = number of independent experiments: Ctrl (11–12/2), VO (10/2), Iso (9–12/2), Iso + VO (7–8/2). Human iPSC-CMs were differentiated from iPSC lines created from a healthy donor and cultured in a medium containing a normal (Ng, 11 mM glucose) or high (HG, 22 mM glucose) concentration of glucose for 7 days. Data are presented as box plots with whiskers showing minimum to maximum values, median, and interquartile range. According to D’Agostino Pearson test, a normal distribution was assumed for **E**: conduction velocity, whereas a non-normal distribution was assumed for **B**, **D**, **E**: FPD_C_, beating rate. *P* values were determined by Bonferroni test after a hierarchical model. *p < 0.05, **p < 0.01, ***p < 0.001. **F** Schematic illustration of the CNP/vosoritide-activated cGMP/cAMP crosstalk in cardiomyocytes (created using BioRender.com)

Finally, we tested whether VO exerts anti-arrhythmic effects in human cardiomyocytes. Therefore, hiPSC-CMs were cultured under high glucose conditions (22 mM glucose, HG) to mimic a diabetic environment [25], whilst hiPSC-CMs cultured in medium with standard glucose conditions (11 mM glucose, NG) served as control (Fig. 7A). Stimulation of the cells with Iso increased CaSp frequency and SR Ca^2+^ leak similarly in both NG and HG hiPSC-CMs. Remarkably, VO was potent in reducing Iso-induced spontaneous Ca^2^⁺ releases in HG but not in NG hiPSC-CMs, mirroring the observations in adult murine cardiomyocytes (Fig. 7B, Supplementary Fig. S5). Co-incubation with the PDE2 inhibitor BAY abolished the anti-arrhythmogenic effect of VO in hiPSC-CMs cultured under HG conditions (Fig. 7C, D). Multi-electrode array recordings further revealed that VO did not affect the beating rate-corrected field potential duration (FPDc), which reflects cardiac repolarisation, nor the conduction velocity or beating rate, in spontaneously beating HG hiPSC-CM cultures, regardless of β-adrenergic stimulation with Iso (Fig. 7D).

Overall, VO reduced Iso-induced pro-arrhythmic Ca^2^⁺ signals—including CaSp, I_Ca,L_ amplitude and SCW—at the cellular level and decreased arrhythmia following I/R at the organ level in the STZ-induced diabetic mouse model. This anti-arrhythmic effect was recapitulated in hiPSC-CMs cultured under high glucose conditions (Supplementary Fig. S6). Mechanistically, VO-induced cGMP generation in cardiomyocytes exposed to hyperglycaemic conditions enhances PDE2 activity, lowers intracellular cAMP, reduces CaMKII activity and consequently diminishes spontaneous RyR2-mediated pro-arrhythmic Ca^2^⁺ releases (Fig. 7F).

## Discussion

Life-threatening arrhythmias are frequent in diabetic patients, particularly after previous sympathetic-activating ischaemic events [12]. In this study, we provide evidence for a novel therapeutic strategy to reduce arrhythmias in STZ-induced diabetic mice. We demonstrate that the clinically approved CNP analogue vosoritide (VO) exerts anti-arrhythmic effects via cGMP-dependent activation of PDE2 after I/R in ex vivo perfused hearts from diabetic mice. At the cellular level, VO-mediated PDE2 stimulation reduced β-AR-mediated arrhythmogenic Ca^2+^ releases, whereas pharmacological inhibition or cardiac-specific deletion of PDE2 abolished the anti-arrhythmic effects of VO. In hiPSC-CMs, VO reduced RyR2-mediated Ca^2+^ leak upon β-adrenergic stimulation. Mechanistically, a reduction of intracellular cAMP as well as a decreased phosphorylation of CaMKII and its target proteins were identified. Thus, our findings suggest that VO merits further investigation for potential repurposing as an anti-arrhythmic drug modulating PDE2 activity.

### Cardiac effects of vosoritide via the NPR-B/cGMP/PDE2 axis

We have previously demonstrated that CNP markedly reduced the number of ventricular arrhythmic events in mice following repeated isoprenaline (Iso) injections. Moreover, CNP attenuated the generation of ventricular arrhythmia in rats with AMI and in isolated murine hearts subjected to I/R injury [5, 14]. Genetic deletion of the CNP-receptor NPR-B increased susceptibility to atrial fibrillation in mice [9]. In addition, CNP has been shown to reduce infarct size following I/R, whilst preserving coronary blood flow and cardiac pressure [15]. Chronic CNP perfusion substantially prevented functional cardiac impairments induced by pressure overload in wild-type mice [24]. Collectively, these studies support our findings that the CNP analogue VO reduces I/R-induced arrhythmia and may confer additional cardioprotective effects following ischaemic events.

The underlying mechanism involves VO-induced cGMP generation and the subsequent activation of the cGMP-dependent PDE2, thereby initiating the negative crosstalk between cGMP and cAMP signalling pathways [48]. As previously shown for CNP in healthy mice [5], VO markedly reduced intracellular cAMP levels via PDE2 in cardiomyocytes from diabetic animals. Notably, in cardiomyocytes from mice with TAC-induced HF, PDE2 overexpression was able to restore disturbed cAMP compartmentalisation [30]. In the present study, VO-mediated PDE2 activation in cells from diabetic mice resulted in a relevant decrease in CaMKII phosphorylation and the CaMKII-dependent phosphorylation of RyR2 and PLB. Consequently, VO substantially reduced pro-arrhythmic Ca^2+^ releases and Ca^2+^ currents following β-adrenergic stimulation, consistent with previous observations using CNP or cGMP [5, 36, 44]. Importantly, these effects of CNP or VO were not observed in PDE2 KO cells.

### Limitations

The effects of VO may differ in humans with diabetes mellitus compared to mice with STZ-induced diabetes. Differences in expression levels of PDE2 and other PDEs, their remodelling under diabetic conditions, and the presence of additional comorbidities, such as obesity and hypertension, should be considered when interpreting the reported data. Our experiments in hiPSC-CMs suggest that the anti-arrhythmic effects of VO might be comparable in murine and in human cells exposed to high glucose levels. Potential contributions from other pathophysiological conditions, such as insulin resistance and lipid accumulation, should also be considered. Our study demonstrates that vosoritide/cGMP-induced PDE2 activation modulates acute pro-arrhythmic Ca^2+^ events in diabetic cardiomyopathy induced by β-adrenergic stress and I/R, but does not address prevention or reversal of cardiac remodelling of Ca^2+^ cycling proteins in diabetic cardiomyopathy. Further research with chronic VO treatment is needed to assess long-term effects.

### Clinical implications

Approved for the treatment of children with achondroplasia and applied subcutaneously, VO is generally well tolerated. Hypotonia may occur during therapy [8]. However, a moderate reduction in blood pressure might be a beneficial side effect in older patients with cardiovascular disease. VO did not affect HR or QT interval duration in ex vivo perfused diabetic Hearts. In clinical phase II and phase III studies, VO was administered once daily subcutaneously to children aged between 5 and 18 years and did not have a clinically meaningful effect on HR after 6 and 12 months of treatment [6]. Although the typical plasma concentration of VO used in achondroplasia therapy is lower than that used here [6], other studies in mice with a maximal plasma concentration of 200 nM have shown that decreases in blood pressure and increases in heart rate remain within acceptable limits (-10 mM Hg, + 50 bpm) [47]. Considering the challenges of anti-arrhythmic therapy and the substantial costs and timeline of de novo drug development, repurposing VO, a compound with safety studies accomplished in children, represents a promising approach for the treatment of arrhythmia [33, 38]. Nevertheless, dose-finding and safety studies for a potential anti-arrhythmic therapy in adults will be essential.

## Conclusion

The present study provides evidence for anti-arrhythmic effects of VO in hearts of diabetic individuals via cGMP-dependent stimulation of PDE2. Stimulation of isolated hearts and cardiomyocytes from diabetic mice with the CNP analogue VO decreased β-adrenergic stimulation-induced phosphorylation levels of pro-arrhythmic targets, leading to a substantial reduction in acute arrhythmogenic events, as of ex vivo arrhythmia, Ca^2+^ sparks, Ca^2+^ fluxes, and spontaneous Ca^2+^ waves. Given the very limited availability of effective pharmacotherapeutic options to treat diabetes-related cardiac arrhythmia, VO/cGMP-mediated PDE2 activation represents a promising mechanism that deserves further preclinical and clinical investigation.

## Supplementary Information

Below is the link to the electronic supplementary material.

Supplementary file1 (PDF 691 KB)

## Data Availability

The data that support the findings of this study are available from the corresponding author, SK, upon reasonable request.

## Abbreviations

AMI: Acute myocardial infarction
ANP: Atrial natriuretic peptide
AP: Action potential
ARNI: Angiotensin receptor-neprilysin inhibitors
BNP: Brain natriuretic peptide
BAY: PDE2-specific inhibitor BAY 60-7550
β-AR: β-Adrenoreceptor
CaMKII: Ca^2+^/calmodulin-dependent kinase II
cAMP: Cyclic adenosine monophosphate
CaSp: Ca^2+^ spark(s)
CaSpF: Ca^2+^ spark frequency
cGMP: Cyclic guanosine monophosphate
CNP: C-type natriuretic peptide
Con: Control (cardiomyocytes or hearts from vehicle-treated mice)
Ctrl: Control group (incubation with buffer or medium only)
DCM: Dilated cardiomyopathy
DiabDCM: Diabetic dilated cardiomyopathy (with insulin-requiring diabetes)
ECG: Electrocardiogram
FAS: Fractional area shortening
FPD_C_: Corrected field potential duration
GAPDH: Glyceraldehyde 3-phosphate dehydrogenase
HbA1c: Glycated haemoglobin A1c
HF: Heart failure
HG: High glucose (22 mM glucose)
hiPSC-CM: Human-induced pluripotent stem cell-derived cardiomyocyte
HR: Heart rate
I/R: Ischaemia/reperfusion
i.p.: Intraperitoneal
iPSC: Induced pluripotent stem cell
Iso: Isoprenaline
KO: Knockout
LAD: Left anterior descending coronary artery
LVEF: Left ventricular ejection fraction
MEA: Multi-electrode array
NF: Non-failing
NG: Normal glucose (11 mM glucose)
NP: Natriuretic peptide
NPR: Natriuretic peptide receptor
PDEs: Phosphodiesterases
PDE2: Phosphodiesterase 2
PLB: Phospholamban
PKA: Protein kinase A
RyR: Ryanodine receptor
SCD: Sudden cardiac death
SCW: Spontaneous Ca^2+^ wave(s)
SR: Sarcoplasmic reticulum
STZ: Streptozotocin
T1DM: Type 1 diabetes mellitus
VES: Ventricular extrasystole
VO: Vosoritide
WT: Wild-type
