# Publisher Correction: The CNP analogue vosoritide mediates PDE2-sensitive anti-arrhythmogenic effects in mouse hearts with STZ-induced type 1 diabetes

**DOI:** 10.1007/s00395-026-01175-8

**Published:** 2026-04-27

**Authors:** Rebecca Firneburg, Katharina Tergau, Eleder Cachorro, Mario Schubert, Anindita Dhara, Xiaojing Luo, Erik Klapproth, Kaomei Guan, Ali El-Armouche, Susanne Kämmerer

**Affiliations:** Medical Faculty, Institute of Pharmacology and Toxicology, Technische Universität Dresden, Fetscherstraße 74, 01307 Dresden, Germany

**Publisher Correction: Basic Research in Cardiology (2025) 120:1173–1191** 10.1007/s00395-025-01141-w

During the production phase of this article contrasts of Figs. 1, 2 and 5 were modified in the typesetting process to a degree that Western blot scans were not displayed as submitted by the authors.

The authors informed the publisher of the typesetting error the day after online publication of the article. The publisher apologizes for the delayed update of the original article and publication of this Publisher Correction.

Figures 1B and 1G were displayed with incorrect gray values:

**Fig. 1:**
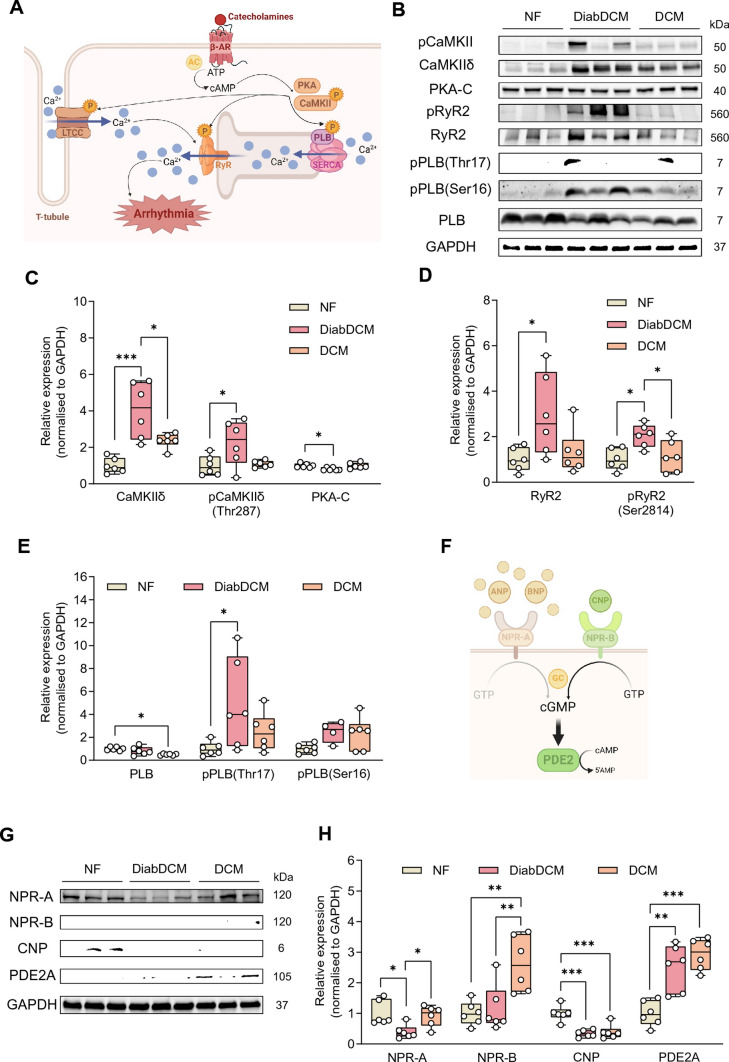
Pro-arrhythmogenic remodelling in patients with dilated cardiomyopathy with (DiabDCM) or without (DCM) insulin-requiring diabetes compared with non-failing controls (NF). **A** Illustration of proteins involved in β-adrenoreceptor (β-AR) signalling and pro-arrhythmogenic remodelling (created using BioRender.com). **B** Representative Western blots and quantification of the indicated protein expression and phosphorylation: **C** Ca^2+^/calmodulin-dependent kinase II (CaMKII) and its phosphorylation at threonine 287 (pCaMKII-Thr287), protein kinase A, catalytic subunit (PKA-C), **D** ryanodine receptor type 2 (RyR2) and its phosphorylation at serine 2814 (pRyR2-Ser2814), **E** phospholamban (PLB) and its phosphorylation at threonine 17 (pPLB-Thr17), and at serine 16 (pPLB-Ser16). **F** Schematic illustrating modulation of cAMP levels by natriuretic peptides via cGMP-dependent stimulation of phosphodiesterase 2 (PDE2) (created using BioRender.com). **G** Representative Western blots and quantification of indicated protein expression: **H** natriuretic peptide receptor A (NPR-A), natriuretic peptide receptor B (NPR-B), C-type natriuretic peptide (CNP), cGMP-dependent PDE2, *N* = 4–6 per group. Protein expressions were normalised to GAPDH, phosphorylation was normalised to total protein. Data are presented as box plots with whiskers showing minimum to maximum values, median and interquartile range. According to D’Agostino Pearson test, a normal distribution was assumed for all data.* P* values were determined by one-way ANOVA followed by Šídák’s multiple comparisons test. *p < 0.05, **p < 0.01, ***p < 0.001

Figure 1 with correct values in sub-figures 1B and 1G is shown below:

**Fig. 1:**
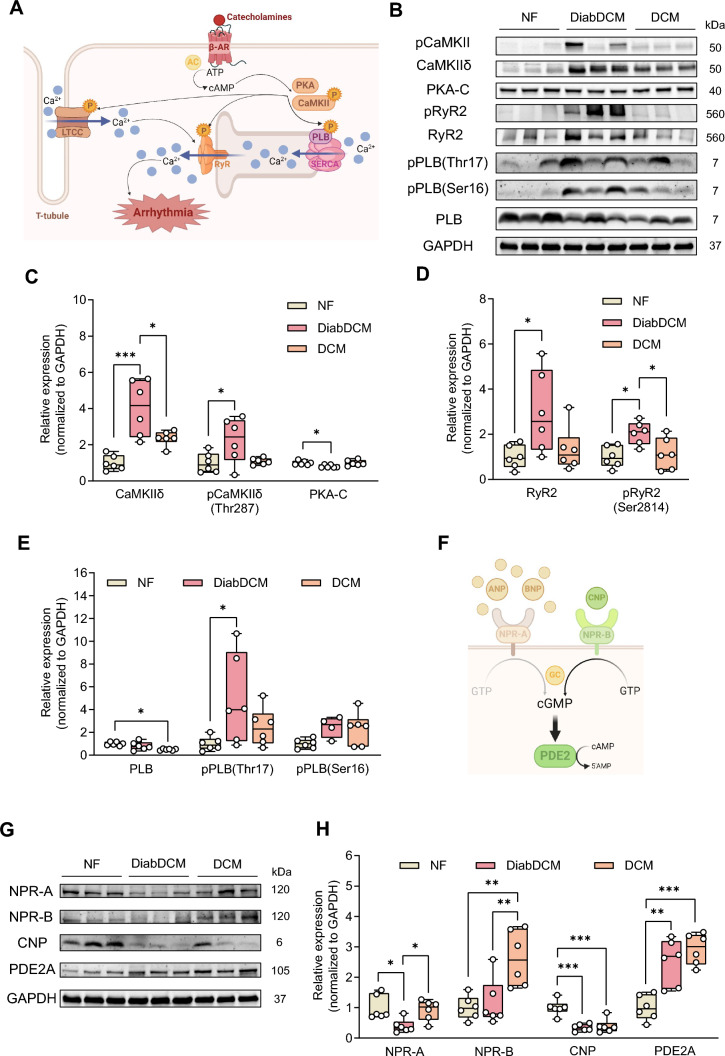
Pro-arrhythmogenic remodelling in patients with dilated cardiomyopathy with (DiabDCM) or without (DCM) insulin-requiring diabetes compared with non-failing controls (NF). **A** Illustration of proteins involved in β-adrenoreceptor (β-AR) signalling and pro-arrhythmogenic remodelling (created using BioRender.com). **B** Representative Western blots and quantification of the indicated protein expression and phosphorylation: **C** Ca^2+^/calmodulin-dependent kinase II (CaMKII) and its phosphorylation at threonine 287 (pCaMKII-Thr287), protein kinase A, catalytic subunit (PKA-C), **D** ryanodine receptor type 2 (RyR2) and its phosphorylation at serine 2814 (pRyR2-Ser2814), **E** phospholamban (PLB) and its phosphorylation at threonine 17 (pPLB-Thr17), and at serine 16 (pPLB-Ser16). **F** Schematic illustrating modulation of cAMP levels by natriuretic peptides via cGMP-dependent stimulation of phosphodiesterase 2 (PDE2) (created using BioRender.com). **G** Representative Western blots and quantification of indicated protein expression: **H** natriuretic peptide receptor A (NPR-A), natriuretic peptide receptor B (NPR-B), C-type natriuretic peptide (CNP), cGMP-dependent PDE2, *N* = 4–6 per group. Protein expressions were normalised to GAPDH, phosphorylation was normalised to total protein. Data are presented as box plots with whiskers showing minimum to maximum values, median and interquartile range. According to D’Agostino Pearson test, a normal distribution was assumed for all data.* P* values were determined by one-way ANOVA followed by Šídák’s multiple comparisons test. *p < 0.05, **p < 0.01, ***p < 0.001

Figure 2A was displayed with incorrect gray values:

**Fig. 2:**
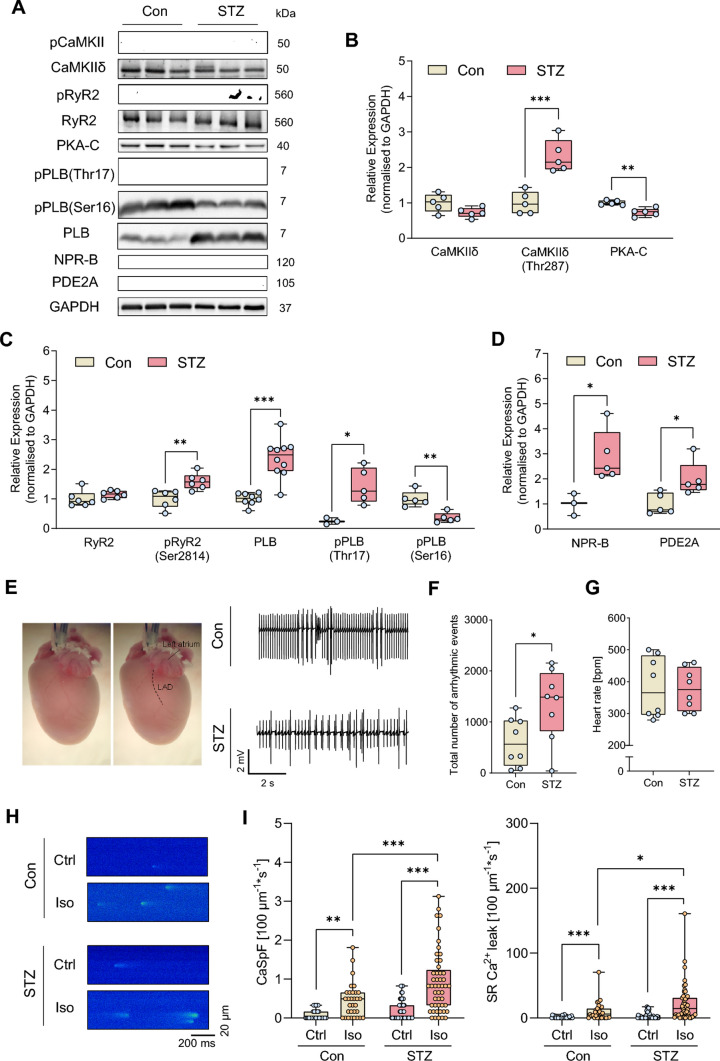
Compared with controls (Con), hearts and cardiomyocytes from mice with STZ-induced diabetes (STZ) exhibit pro-arrhythmogenic remodelling and increased arrhythmia susceptibility following ischaemia–reperfusion injury (I/R) or β-adrenergic stimulation. **A** Representative Western blots and quantification of indicated protein expression and phosphorylation in isolated ventricular cardiomyocytes from Con and STZ: **B** Ca^2+^/calmodulin-dependent kinase II (CaMKII) and its phosphorylation at threonine 287 (pCaMKII-Thr287), protein kinase A, catalytic subunit (PKA-C), **C** ryanodine receptor type 2 (RyR2) and its phosphorylation at serine 2814 (pRyR2-Ser2814), phospholamban (PLB) and its phosphorylation at threonine 17 (pPLB-Thr17), and serine 16 (pPLB-Ser16), **D** natriuretic peptide receptor B (NPR-B) and phosphodiesterase 2 (PDE2), *N*  = 3–10 per group. Protein expressions were normalised to GAPDH, phosphorylation was normalised to total protein. **E** Representative ECG traces of ex vivo perfused Con and STZ hearts after I/R. **F** Total number of arrhythmic events and **G** mean heart rates (30 min after ex vivo I/R), *N* = 8 per group. **H** Representative Ca^2+^ spark (CaSp) recordings from Con and STZ cardiomyocytes under basal conditions (Ctrl), or following stimulation with Iso (10 nM) for 7 min and pacing at 1 Hz, 10 mV for 10 s. **I** Quantification of CaSp frequency (CaSpF) and SR Ca.^2+^ leak upon the respective conditions. *n*  =  number of cells / *N* = number of animals: Con: Ctrl (30/4), Iso (31/4); STZ: Ctrl (49/7), Iso (49/7). Data are presented as box plots with whiskers showing minimum to maximum values, median and interquartile range. According to D’Agostino Pearson test, a normal distribution was assumed for **B**, **C**: pRyR2-Ser2814, PLB, pPLB-Thr17, pPLB-Ser16, **D** and **F**, whereas a non-normal distribution was assumed for **C**: RyR2, **G**, and **I**. *P* values were determined by *t* test with (**C**: PLB) or without Welch’s correction (**B**, **C**: pRyR2-Ser2814, pPLB-Thr17, pPLB-Ser16, **D**, **F**), Mann–Whitney test (**C**: RyR2, **G**) or two-way ANOVA (**I**). *p < 0.05, **p < 0.01, ***p < 0.001

Figure 2 with correct values in sub-figure 2A is shown below:

**Fig. 2:**
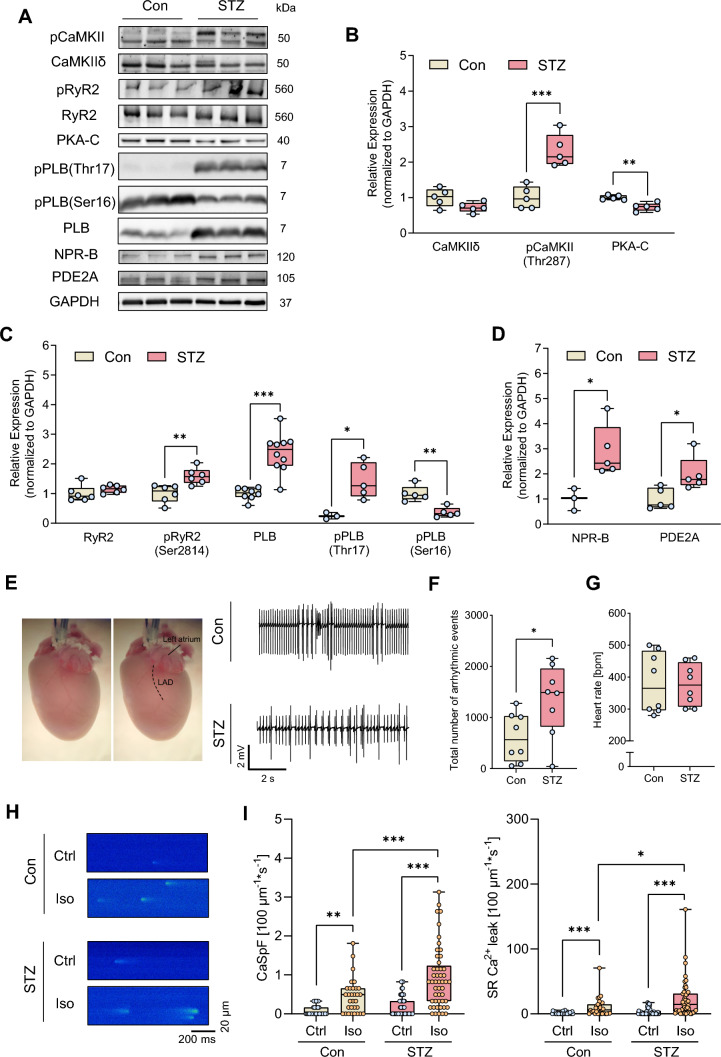
Compared with controls (Con), hearts and cardiomyocytes from mice with STZ-induced diabetes (STZ) exhibit pro-arrhythmogenic remodelling and increased arrhythmia susceptibility following ischaemia–reperfusion injury (I/R) or β-adrenergic stimulation. **A** Representative Western blots and quantification of indicated protein expression and phosphorylation in isolated ventricular cardiomyocytes from Con and STZ: **B** Ca^2+^/calmodulin-dependent kinase II (CaMKII) and its phosphorylation at threonine 287 (pCaMKII-Thr287), protein kinase A, catalytic subunit (PKA-C), **C** ryanodine receptor type 2 (RyR2) and its phosphorylation at serine 2814 (pRyR2-Ser2814), phospholamban (PLB) and its phosphorylation at threonine 17 (pPLB-Thr17), and serine 16 (pPLB-Ser16), **D** natriuretic peptide receptor B (NPR-B) and phosphodiesterase 2 (PDE2), *N*  = 3–10 per group. Protein expressions were normalised to GAPDH, phosphorylation was normalised to total protein. **E** Representative ECG traces of ex vivo perfused Con and STZ hearts after I/R. **F** Total number of arrhythmic events and **G** mean heart rates (30 min after ex vivo I/R), *N* = 8 per group. **H** Representative Ca^2+^ spark (CaSp) recordings from Con and STZ cardiomyocytes under basal conditions (Ctrl), or following stimulation with Iso (10 nM) for 7 min and pacing at 1 Hz, 10 mV for 10 s. **I** Quantification of CaSp frequency (CaSpF) and SR Ca.^2+^ leak upon the respective conditions. *n*  =  number of cells / *N* = number of animals: Con: Ctrl (30/4), Iso (31/4); STZ: Ctrl (49/7), Iso (49/7). Data are presented as box plots with whiskers showing minimum to maximum values, median and interquartile range. According to D’Agostino Pearson test, a normal distribution was assumed for **B**, **C**: pRyR2-Ser2814, PLB, pPLB-Thr17, pPLB-Ser16, **D** and **F**, whereas a non-normal distribution was assumed for **C**: RyR2, **G**, and **I**. *P* values were determined by *t* test with (**C**: PLB) or without Welch’s correction (**B**, **C**: pRyR2-Ser2814, pPLB-Thr17, pPLB-Ser16, **D**, **F**), Mann–Whitney test (**C**: RyR2, **G**) or two-way ANOVA (**I**). *p < 0.05, **p < 0.01, ***p < 0.001

Figure 5E was displayed with incorrect gray values:

**Fig. 5:**
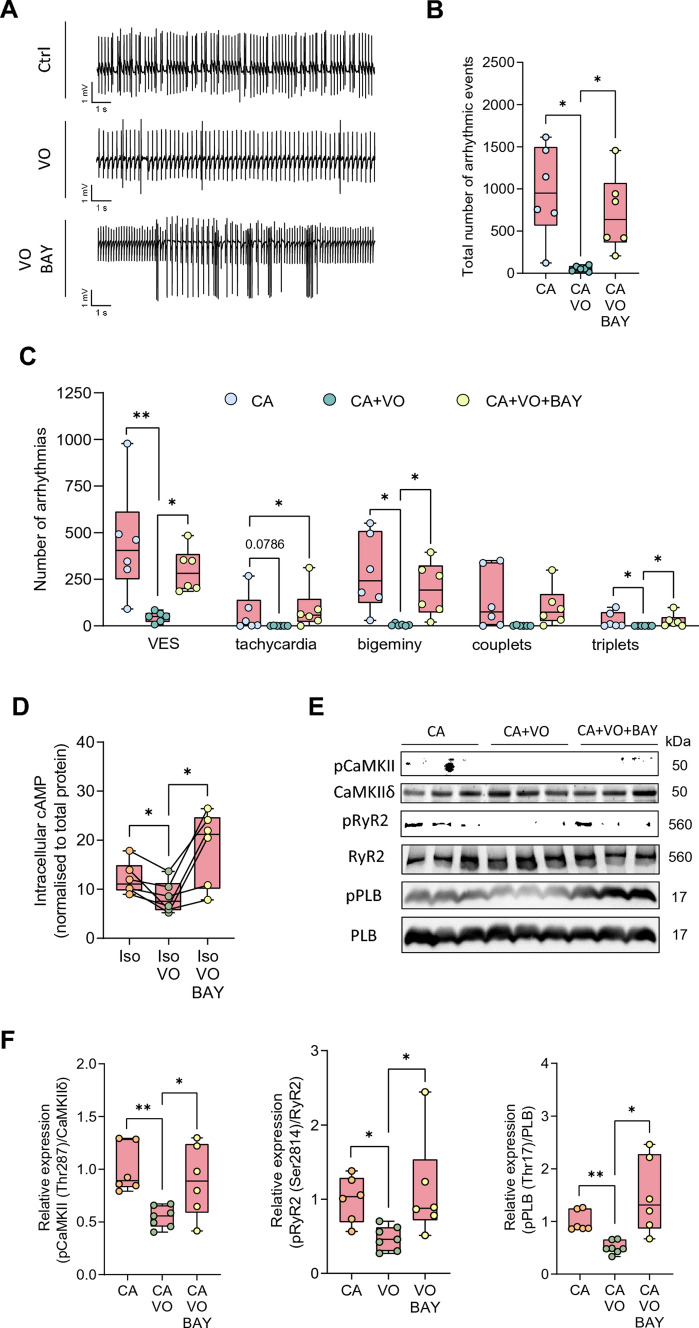
Vosoritide (VO) protects hearts from diabetic mice against arrhythmia following ex vivo ischaemia/reperfusion injury (I/R) by stimulating PDE2 and reducing cAMP/CaMKII-dependent phosphorylation of Ca^2+^ cycling proteins at the cellular level.** A** Representative ECG traces, **B** total number of arrhythmic events, **C** number of ventricular extrasystoles (VES), tachycardia, bigeminy, couplets and triplets, in hearts from STZ-treated mice perfused ex vivo with Krebs–Henseleit buffer containing physiological catecholamine (CA) concentrations (10 nM norepinephrine, 3.5 nM epinephrine) with or without vosoritide (200 nm), or BAY 60-7550 (BAY, 300 nm) after I/R, *N*  = 6 per group. **D** cAMP content in isolated ventricular cardiomyocytes quantified by direct cAMP ELISA following treatment with Iso (10 nM), Iso + VO (1 µM), or Iso + VO + BAY (100 nM) for 10 min, *N*  = 6 per group. Relative cAMP concentrations were normalised to corresponding sample protein content and the Iso group. **E** Representative Western blots, and **F** quantification of Ca^2+^/calmodulin-dependent kinase II (CaMKII) phosphorylation at threonine 287 (pCaMKII-Thr287) and CaMKII-dependent phosphorylation of ryanodine receptor 2 at serine 2814 (pRyR2-Ser2814) and phospholamban at threonine 17 (pPLB-Thr17) in murine ventricular heart tissue following ex vivo Langendorff perfusion with Krebs–Henseleit buffer containing catecholamines (CA, 10 nM norepinephrine, 3.5 nM epinephrine), CA + VO (200 nM), or CA + VO + BAY (300 nM) for 1:15 h, *N* = 6 (CA, CA + VO + BAY), or *N* = 7 (CA + VO) per group. Protein phosphorylation was normalised to total protein. Data are presented as box plots with whiskers showing minimum to maximum values, median, and interquartile range. According to D’Agostino Pearson test, normal distribution was assumed for **B**, **C**: bigeminy, couplets, **D**, **F**: pCaMKII-Thr287, pPLB-Thr17, non-normal distribution was assumed for **C**: VES, tachycardia, triplets and **F**: pRyR2-Ser2814. *p* values were determined by Dunnett’s T3 multiple comparisons test after Brown–Forsythe test and Welch ANOVA (**B**, **C**: bigeminy, F: pPLB-Thr17), Šídák’s multiple comparisons test after one-way ANOVA (**C**: couplets, **F**: pCaMKII-Thr287), RM one-way ANOVA (**D**), or Dunn’s multiple comparisons test after Kruskal–Wallis test (**C**: VES, tachycardia, triplets, **F**: pRyR2-Ser2814). *p < 0.05, **p < 0.01

Figure 5 with correct values in sub-figure 5E is shown below:

**Fig. 5:**
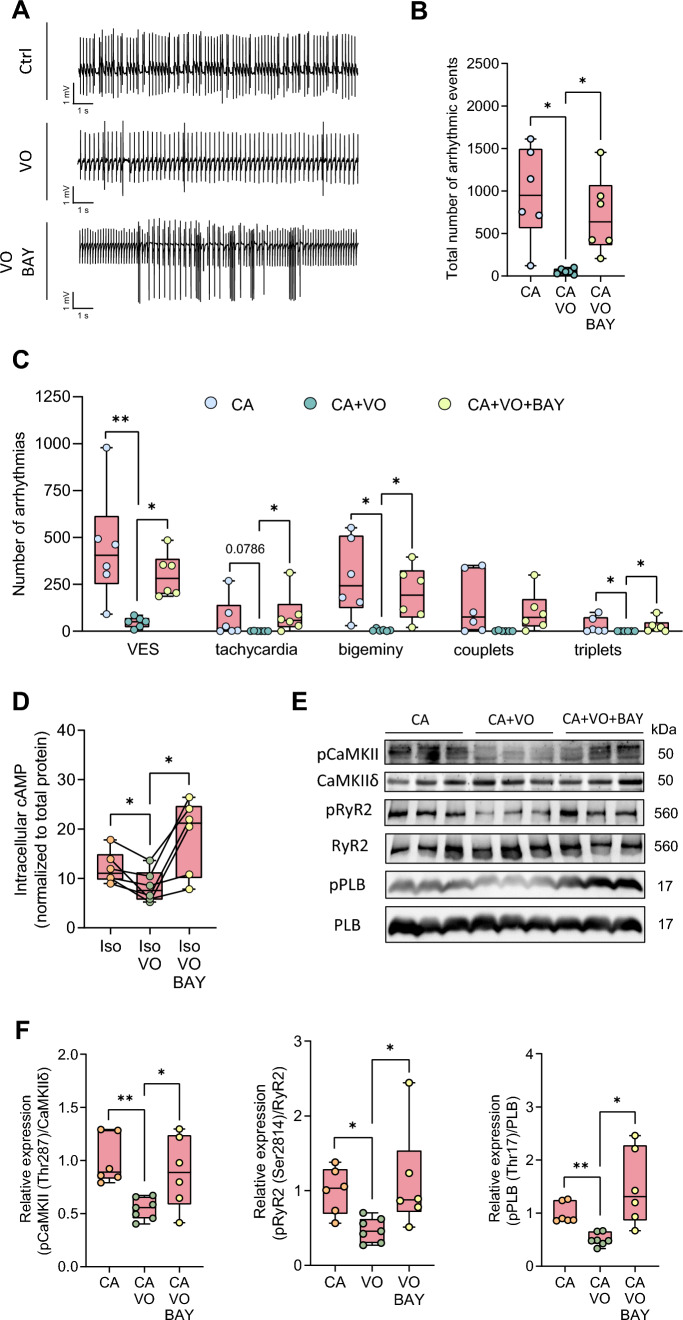
Vosoritide (VO) protects hearts from diabetic mice against arrhythmia following ex vivo ischaemia/reperfusion injury (I/R) by stimulating PDE2 and reducing cAMP/CaMKII-dependent phosphorylation of Ca^2+^ cycling proteins at the cellular level.** A** Representative ECG traces, **B** total number of arrhythmic events, **C** number of ventricular extrasystoles (VES), tachycardia, bigeminy, couplets and triplets, in hearts from STZ-treated mice perfused ex vivo with Krebs–Henseleit buffer containing physiological catecholamine (CA) concentrations (10 nM norepinephrine, 3.5 nM epinephrine) with or without vosoritide (200 nm), or BAY 60-7550 (BAY, 300 nm) after I/R, *N*  = 6 per group. **D** cAMP content in isolated ventricular cardiomyocytes quantified by direct cAMP ELISA following treatment with Iso (10 nM), Iso + VO (1 µM), or Iso + VO + BAY (100 nM) for 10 min, *N*  = 6 per group. Relative cAMP concentrations were normalised to corresponding sample protein content and the Iso group. **E** Representative Western blots, and **F** quantification of Ca^2+^/calmodulin-dependent kinase II (CaMKII) phosphorylation at threonine 287 (pCaMKII-Thr287) and CaMKII-dependent phosphorylation of ryanodine receptor 2 at serine 2814 (pRyR2-Ser2814) and phospholamban at threonine 17 (pPLB-Thr17) in murine ventricular heart tissue following ex vivo Langendorff perfusion with Krebs–Henseleit buffer containing catecholamines (CA, 10 nM norepinephrine, 3.5 nM epinephrine), CA + VO (200 nM), or CA + VO + BAY (300 nM) for 1:15 h, *N* = 6 (CA, CA + VO + BAY), or *N* = 7 (CA + VO) per group. Protein phosphorylation was normalised to total protein. Data are presented as box plots with whiskers showing minimum to maximum values, median, and interquartile range. According to D’Agostino Pearson test, normal distribution was assumed for **B**, **C**: bigeminy, couplets, **D**, **F**: pCaMKII-Thr287, pPLB-Thr17, non-normal distribution was assumed for **C**: VES, tachycardia, triplets and **F**: pRyR2-Ser2814. *p* values were determined by Dunnett’s T3 multiple comparisons test after Brown–Forsythe test and Welch ANOVA (**B**, **C**: bigeminy, F: pPLB-Thr17), Šídák’s multiple comparisons test after one-way ANOVA (**C**: couplets, **F**: pCaMKII-Thr287), RM one-way ANOVA (**D**), or Dunn’s multiple comparisons test after Kruskal–Wallis test (**C**: VES, tachycardia, triplets, **F**: pRyR2-Ser2814). *p < 0.05, **p < 0.01

The original article has been corrected.

