# Supplementary material for: The CNP analogue vosoritide mediates PDE2-sensitive anti-arrhythmogenic effects in mouse hearts with STZ-induced type 1 diabetes
Source: Basic Res Cardiol. 2025 Sep 18;120(6):1173–91. doi: 10.1007/s00395-025-01141-w (PMC12680885; doi:10.1007/s00395-025-01141-w)
Supplement: Supplementary file 1 — Supplementary file1 (PDF 691 KB) [file 395_2025_1141_MOESM1_ESM.pdf]

Supplemental Material

The CNP analogue vosoritide mediates PDE2-sensitive anti-arrhythmogenic effects in mouse hearts with STZ-induced type 1 diabetes

Rebecca Firneburg[1], Katharina Tergau[1], Eleder Cachorro[1], Mario Schubert[1], Anindita Dhara[1], Xiaojing Luo[1], Erik Klapproth[1], Kaomei Guan[1], Ali El-Armouche[1], Susanne Kämmerer[1]

[1]Institute of Pharmacology and Toxicology, Medical Faculty, Technische Universität Dresden, Germany

Supplementary Figures

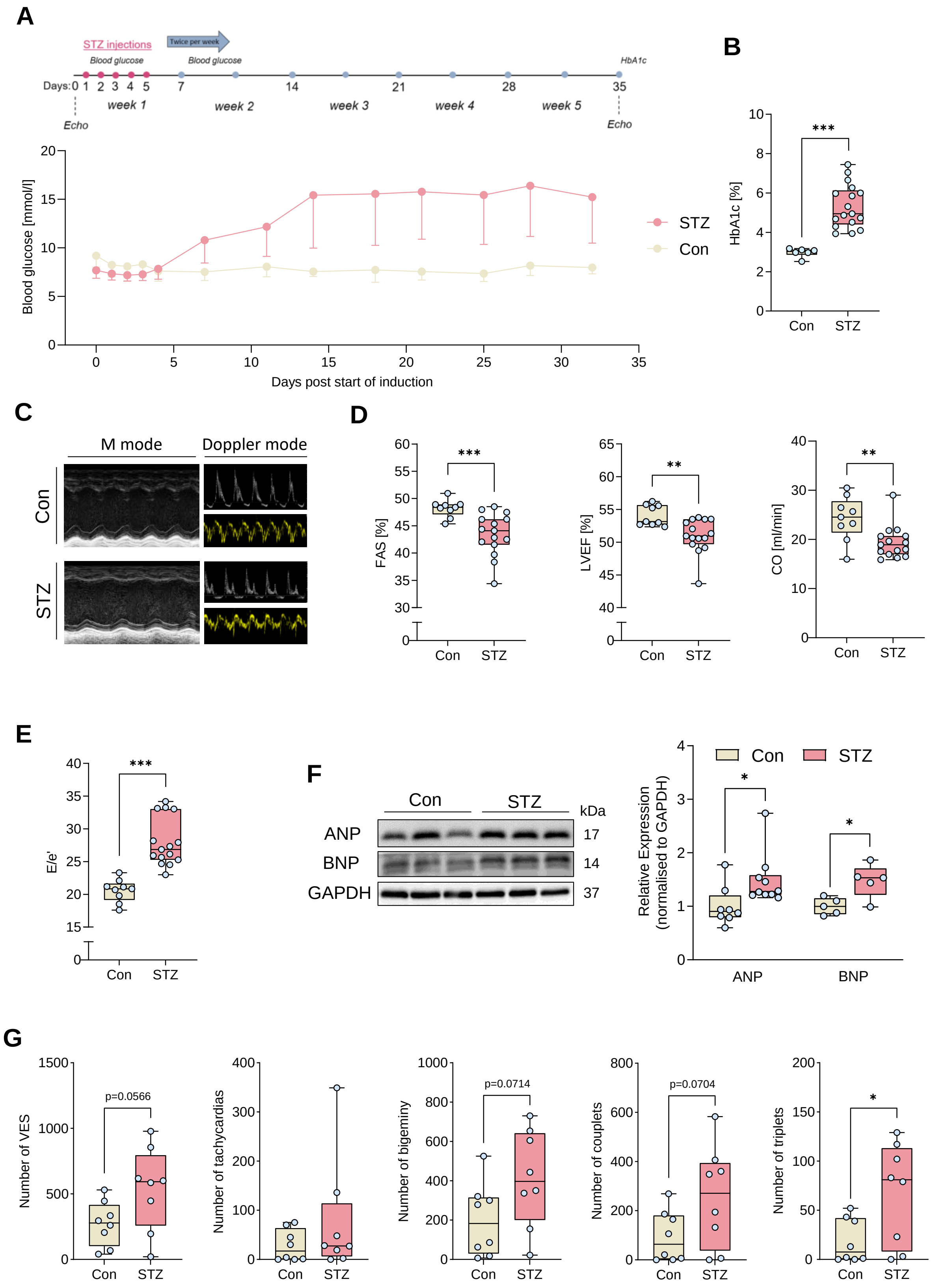

Fig. S1, Diabetes induction leads to cardiac dysfunction and enhanced arrhythmogenesis following ischaemia/reperfusion injury (I/R). **A**, Schematic of diabetes induction and blood glucose levels following 5 consecutive i.p. injections of streptozotocin (STZ, 50 µg/g). **B**, HbA1c levels in control (Con) and STZ-treated (STZ) mice 5 weeks after the start of diabetes induction, *N*=6 (Con) or 17 (STZ). **C**, Original M mode and Doppler mode registrations of STZ and Con hearts representing: **D**, left ventricular ejection fraction (LVEF), fractional area shortening (FAS), and cardiac output (CO), and **E**, E/e' ratios in Con and STZ assessed by echocardiography, *N*=9 (Con) or 15 (STZ). **F**, Protein expression of atrial natriuretic peptide (ANP) and brain natriuretic peptide (BNP) in ventricular cardiomyocytes from Con and STZ, normalised to GAPDH, *N*=5-10. **G**, ventricular extrasystoles (VES), tachycardias, bigeminy, couplets and triplets in *ex vivo* perfused hearts after I/R, *N*=8 per group. Data are presented as box plots with whiskers showing minimum to maximum values, median and interquartile range. According to D'Agostino Pearson test, a normal distribution was assumed for B, D: CO, E, F: BNP, G: VES, bigeminy, couplets, triplets, whereas a non-normal distribution was assumed for D: LVEF, FAS, F: ANP, G: tachycardias. *P* values were determined by *t* test with (B, E, G: triplets) or without (D: CO, F: BNP, G: VES, bigeminy, couplets) Welch's correction or by Mann-Whitney test (D: LVEF, FAS, F: ANP, G: tachycardias). **p*<0.05, ***p*<0.01, ****p*<0.001.

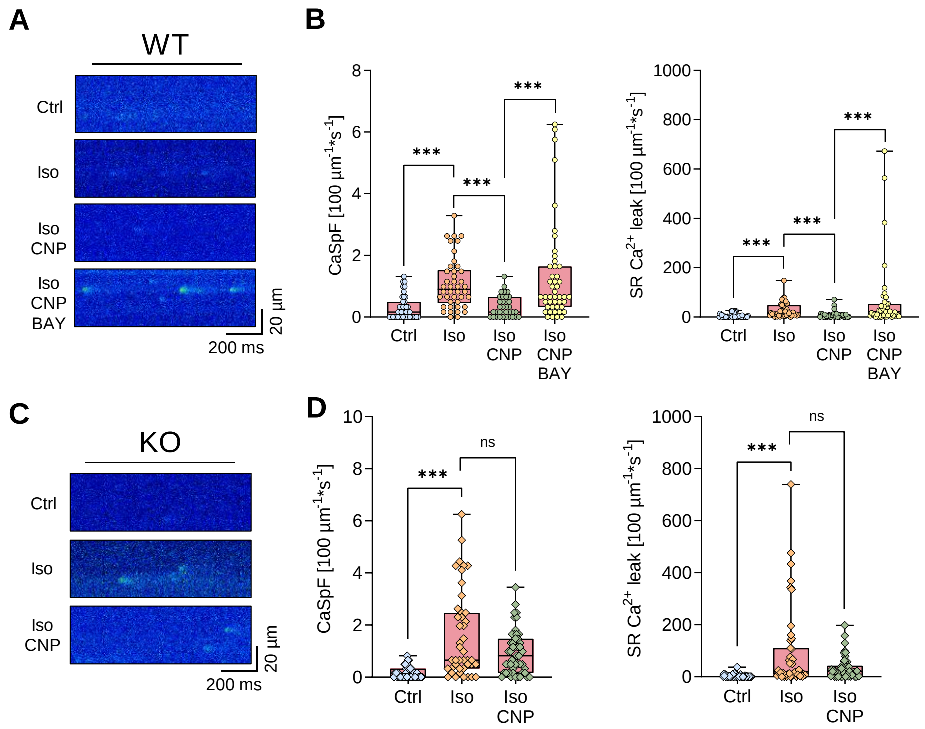

Supplementary Figure 2

Fig. S2, cGMP-dependent PDE2 stimulation with CNP protects ventricular cardiomyocytes isolated from mice with STZ-induced diabetes against pro-arrhythmogenic intracellular Ca^{2+}

fluxes. **A**, Representative Ca^{2+} spark (CaSp) recordings in cells from diabetic wild-type mice under basal conditions (Ctrl), or following stimulation with Iso (10 nM), Iso+CNP (1 µM), or Iso+CNP+BAY (100 nM) for 7 min and pacing at 1 Hz, 10 mV for 10 sec. **B**, Quantification of CaSp frequency (CaSpF) and SR Ca^{2+} leak under the respective conditions, n = number of cells / *N* = number of animals: Ctrl (43/5), Iso (42/5), Iso+CNP (35/5), Iso+CNP+BAY (46/5). **C**, Representative CaSp recordings in cells from diabetic PDE2 knockout (KO) mice under basal conditions (Ctrl), or following stimulation with Iso (10 nM), or Iso+CNP (1 µM) for 7 min and pacing at 1 Hz, 10 mV for 10 sec. **D**, Quantification of Ca^{2+} spark frequency (CaSpF) and SR Ca^{2+} leak under the respective conditions, n = number of cells / *N* = number of animals: Ctrl (47/5), Iso (43/5), Iso+CNP (49/5). Data are presented as box plots with whiskers showing minimum to maximum values, median, and interquartile range. According to D'Agostino Pearson test, all data were assumed to be non-normally distributed. *P* values were determined by Bonferroni test after a hierarchical model. $^{**}p<0.01$, $^{***}p<0.001$.

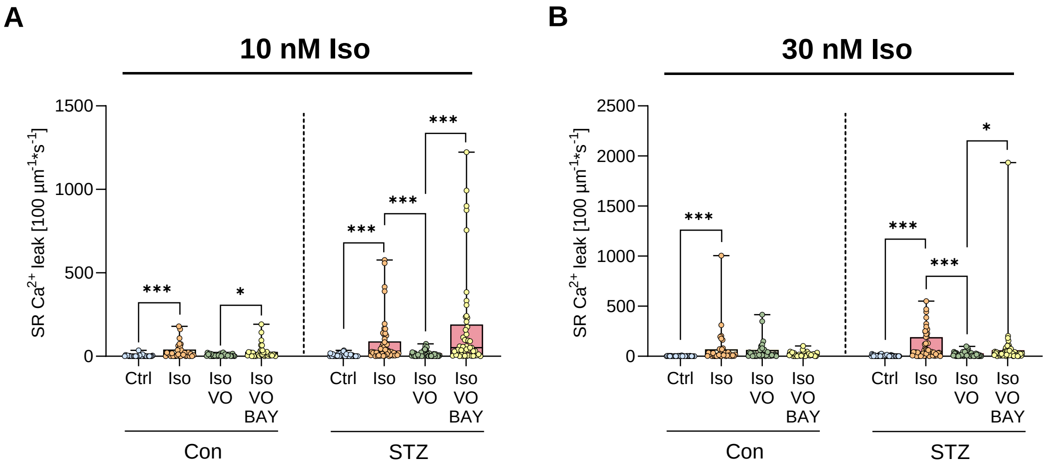

Supplementary Figure 3

Fig. S3, cGMP-dependent PDE2 stimulation with vosoritide (VO) protects ventricular cardiomyocytes isolated from diabetic animals from pro-arrhythmogenic intracellular Ca^{2+} leak. **A**, Quantification of SR Ca^{2+} leak in cardiomyocytes from wild-type mice without (Con) or with STZ-induced diabetes (STZ) under basal conditions (Ctrl), or following stimulation with Iso (10 nM), Iso+VO (1 µM), or Iso+VO+BAY (100 nM) for 7 min and pacing at 1 Hz, 10 mV for 10 sec, n = number of cells / *N* = number of animals: Con: Ctrl (30/4), Iso (33/4), Iso+VO (31/4), Iso+VO+BAY (35/4); STZ: Ctrl (50/6), Iso (50/6), Iso+VO (52/6), Iso+VO+BAY (49/6). **B**, Quantification of SR Ca^{2+} leak in cardiomyocytes from wild-type mice without (Con) or with STZ-induced diabetes (STZ) under basal conditions (Ctrl), or following stimulation with Iso (30 nM), Iso+VO (1 µM), or Iso+VO+BAY (100 nM) for 7 min and pacing at 1 Hz, 10 mV for 10 sec, n = number of cells / *N* =

number of animals: Con: Ctrl (34/4), Iso (35/4), Iso+VO (37/4), Iso+VO+BAY (32/4); STZ: Ctrl (51/7), Iso (49/7), Iso+VO (48/7), Iso+VO+BAY (55/7). Data are presented as box plots with whiskers showing minimum to maximum values, median, and interquartile range. According to D'Agostino Pearson test, all data were assumed to be non-normally distributed. *P* values were determined by Bonferroni test after a hierarchical model. **p*<0.05, ****p*<0.001.

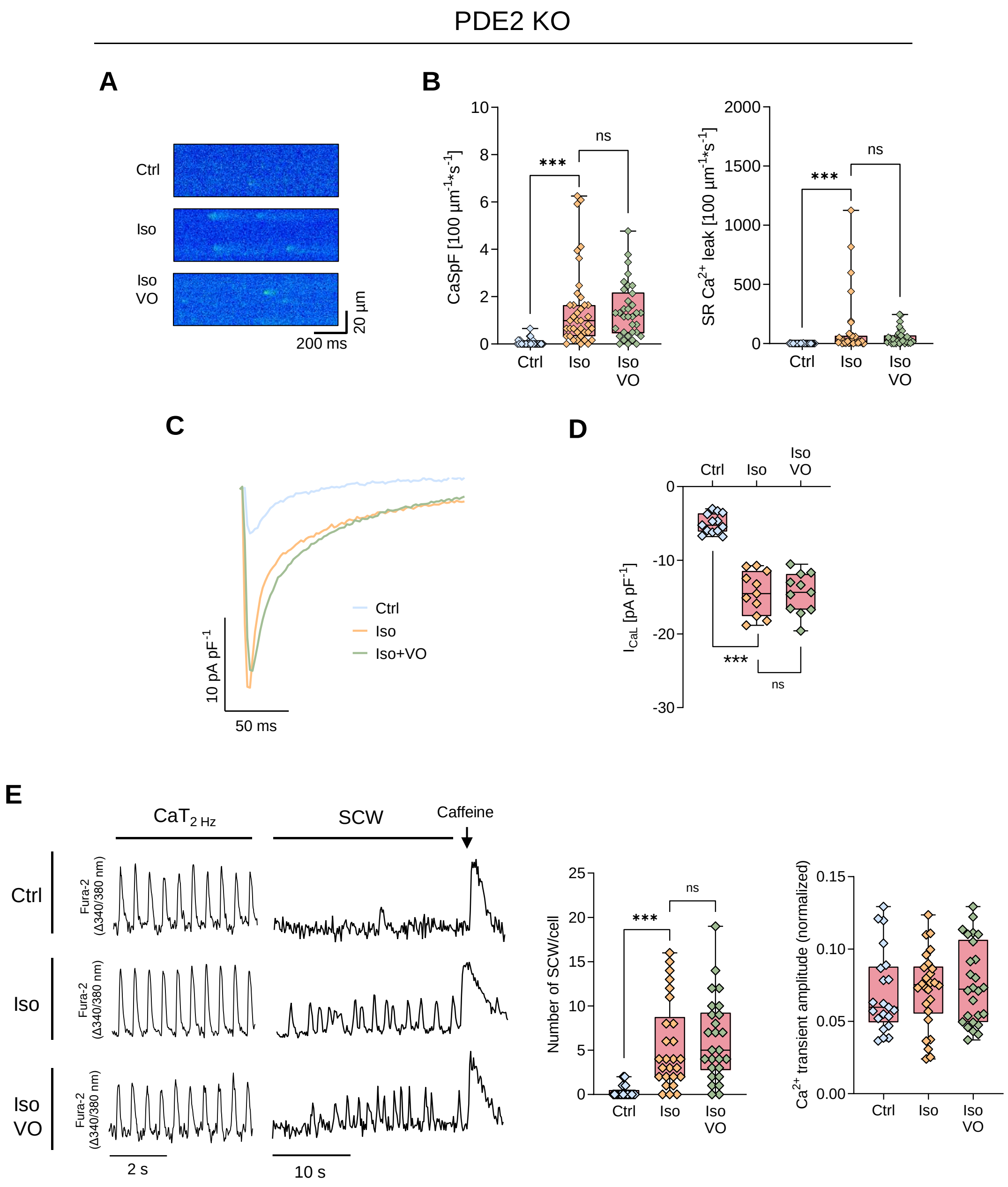

Supplementary Figure 4

Fig. S4, The antiarrhythmic effects of vosoritide (VO) are blunted in ventricular cardiomyocytes isolated from diabetic mice with cardiac-specific PDE2 knockout (KO). **A**, Representative recordings of Ca^{2+} sparks (CaSp) in cells from diabetic PDE2 KO mice under basal conditions

(Ctrl), or following stimulation with Iso (10 nM), or Iso+VO (1 µM) for 7 min and pacing at 1 Hz, 10 mV for 10 sec. **B**, Quantification of CaSp frequency (CaSpF) and SR Ca^{2+} leak under the respective conditions, n = number of cells / *N* = number of animals: Ctrl (34/4), Iso (39/4), Iso+VO (34/4). **C**, Representative $I_{Ca,L}$ recordings in cardiomyocytes from diabetic PDE2 KO mice under basal conditions (Ctrl), or following stimulation with Iso (30 nM), or Iso+VO (10 µM) for 10 min. **D**, $I_{Ca,L}$ current density measured at 0 mV under the respective conditions, n = number of cells / *N* = number of animals: Ctrl (13/9), Iso (11/6), Iso+VO (11/6). **E**, Representative traces of cardiomyocytes from diabetic PDE2 KO cardiomyocytes loaded with Fura-2 under basal conditions (Ctrl), or following stimulation with Iso (30 nM), or Iso+VO (10 µM) for 10 min and subjected to arrhythmia provocation (2 Hz pacing, 30 s); number of spontaneous Ca^{2+} waves (SCW) per cell and Ca^{2+} transient amplitude at 2 Hz under the respective conditions, n = number of cells / *N* = number of animals: Ctrl (21/4), Iso (26/5), Iso+VO (26/5). Data stated as box plots with whiskers showing minimum to maximum values, median, and interquartile range. According to D'Agostino Pearson test, a normal distribution was assumed for D and E: Ca^{2+} transient amplitude, whereas a non-normal distribution was assumed for B, E: SCW. *P* values were determined by Bonferroni test after a hierarchical model. ***p<0.001.

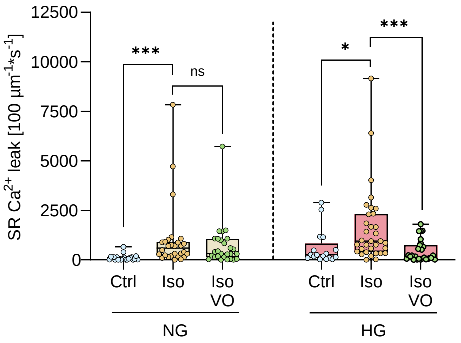

Supplementary Figure 5

Fig. S5, Vosoritide (VO) reduces the SR Ca^{2+} leak in hiPSC-CMs cultured under high glucose (HG) conditions via activation of PDE2. **A**, Quantification of the SR Ca^{2+} leak in hiPSC-CM cultured under normo (NG) or high (HG) glucose conditions under basal conditions (Ctrl), or following stimulation with Iso (100 nM) and Iso+VO (1 µM) for 5 min and pacing at 0.5 Hz, 10 mV, n = number of cells / *N* = number of independent experiments: NG: Ctrl (22/4), Iso (28/4), Iso+VO (27/4), HG: Ctrl (17/3), Iso (33/4), Iso+VO (27/4). Human iPSC-CMs were differentiated from hiPSC lines created of a healthy donor and cultured in a medium containing a standard (NG, 11 mM glucose) or high (HG, 22 mM glucose) concentration of glucose for 7 days. Data are presented as box plots with whiskers showing minimum to maximum values, median, and interquartile range. According to D'Agostino Pearson test, the data were assumed to be non-

normally distributed. *P* values were determined by Bonferroni test after a hierarchical model. *$p<0.05$, **$p<0.01$, ***$p<0.001$.

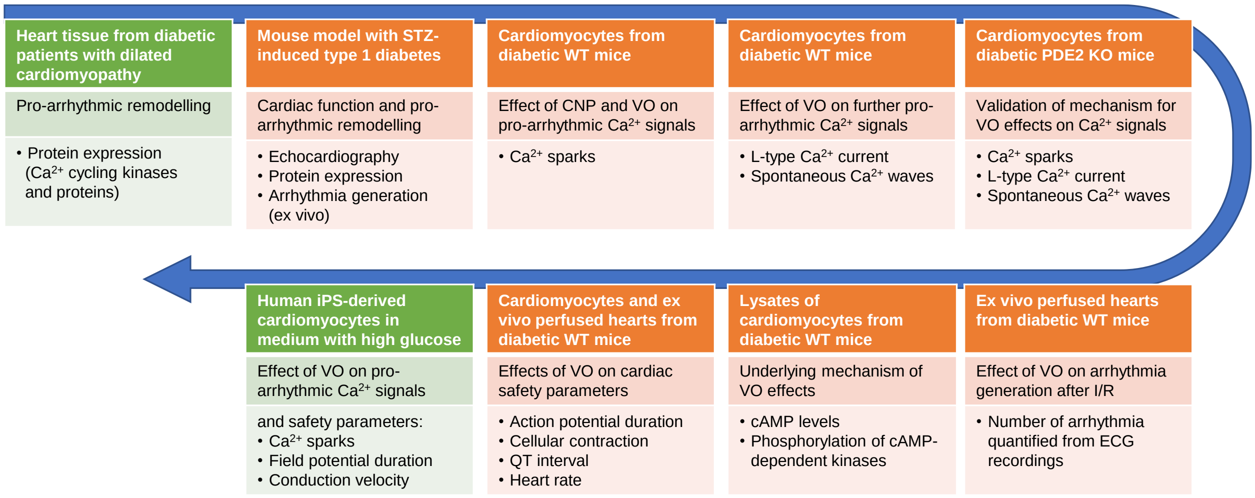

Supplemental Figure 6

Fig. S6, Timeline of the study.

Supplementary Tables

Table S1: Human heart tissue excised from explanted hearts of NYHA III-IV DCM patients or unsuitable donor hearts was used for Western blot analysis. All patients participating in this study were of Caucasian origin and provided written informed consent in accordance with the Declaration of Helsinki. The study was approved by the Research Ethics Committee of TU Dresden (Official approval number: EK 114082202) and the Albert Szent-Gyorgyi Medical University Ethical Review Board (Szeged). The table below summarises the patients' characteristics including sex, disease status and medication.

**Sex**	**Age**	**NYHA**	**Diagnosis**	**Medication**
Patient	[years]			
Male	64	III	DCM	allopurinol, **insulin**, isosorbide dinitrate, metoprolol, ramipril, torasemide,
Male	62	III-IV	DCM	carvedilol, digitoxin, (disopyrine/ disopyramide), enalapril, enoximone, **insulin**
Male	60	III-IV	DCM	allopurinol, digitoxin, **insulin**, ramipril, spironolactone,
Male	56	IV	DCM	amiodarone, digitoxin, enoxaparine, eplerenone, escitalopram, esomeprazole, **insulin**, L-thyroxine, metoprolol, phenprocoumon, ramipril, torasemide/ xipamide
Male	54	IV	DCM	amiodarone, carvedilol, digitoxin, dobutamine, enoximone, esomeprazole, ezetimibe, heparin, hydrochlorotiazide, **insulin**, simvastatin, torasemide, valsartan
Male	38	III-IV	DCM	allopurinol, atorvastatin, carvedilol, digitoxin, dobutamine, enoximone, **insulin**, torasemide/xipamide
Male	75	III	DCM	carvedilol, phenprocoumon
Male	68	III	DCM	digitoxin, metoprolol, ramipril, simvastatin
Male	64	III	DCM	metoprolol, pantoprazole, ramipril, phenprocoumon, torasemide
Male	63	III	DCM	carvedilol, pantoprazole, ramipril
Male	58	III	DCM	cadesartan, digitoxin, fodaparinux metoprolol, hydrochlorotiazide, pentaerythritol tetranitrate

Male	55	III	DCM	metoprolol, ramipril
Donor				
Female	58	-	-	
Male	52	-	-	
Female	49	-	-	
Male	38	-	-	
Male	38	-	-	
Male	25	-	-	

Table S2: Characteristics of mouse lines including sources, genetical background, identity numbers and other information.

	Species	Vendor or Source	Back-ground Strain	Other Information	Persistent ID / URL (RRID)
**Parent – Male 1**	Mus musculus	EC TU-DRESDEN	C57BL/6N	Cardiac-specific PDE2 knockout: Cre-recombinase under the control of α-myosin heavy chain promoter; floxed PDE2A exon 4 (PDE2 KO)	PDE2_flox_aMHC_Cre
**Parent – Female 1**	Mus musculus	EC TU-DRESDEN	C57BL/6N	Floxed PDE2A exon 4 ($PDE2^{fl/fl}$)	PDE2_flox_aMHC_Cre
**Parent – Male 2**	Mus musculus	EC TU-DRESDEN	C57BL/6J	Wild-type (WT)	C57BL/6J (RRID: IMSR_RJ:C57BL-6NRJ)
**Parent – Female 2**	Mus musculus	EC TU-DRESDEN	C57BL/6J	Wild-type (WT)	C57BL/6J (RRID: IMSR_RJ:C57BL-6NRJ)

Table S3: Resources of antibodies.

Antibody	Catalogue no.	RRID	Supplier	Dilution
ANP	sc-18811	AB_2155322	Santa Cruz	1:200
BNP (NPPB)	abx001791	-	Abbexa	1:200
CaMKIIδ,	MAB4176	AB_2068084	R&D Systems	1:500
CaMKII pThr286	MA1-047	AB_325402	Thermo,	1:1,000
CNP	PA5-103119	AB_2852489	Invitrogen	1:500
GAPDH	sc-365062	AB_10847862	Santa Cruz	1:1,000
NPR-A	ab14356	AB_2283086	Abcam	1:500
NPR-B	PGCB-201AP	AB_2315120	FabGennix	1:500
PDE2A	PDE2A-101AP	-	FabGennix	1:500

PKA-C	610981	AB_398294	BD Bioscience	1:3,000
PLB	A010-14	AB_2617049	Badrilla	1:2,000
PLB pSer16,	A010-12	AB_2617047	Badrilla	1:2,000
PLB pThr17	A010-13	AB_2617048	Badrilla	1:2,000
RyR2	HPA020028	AB_1856528	Sigma-Aldrich	1:2,000
RyR2 pSer2814	A010-31AP	AB_3665192	Badrilla	1:5,000
Goat anti-mouse HRP	A3682	AB_258100	Sigma-Aldrich	1:10,000
Goat anti rabbit-HRP	A0545	AB_257896	Sigma-Aldrich	1:10,000
Donkey anti goat-HRP	sc-2020	AB_631728	Santa Cruz	1:5,000

Table S4: Resources of chemicals.

Chemical	IUPAC name	Catalogue no.	Supplier
BAY 60-7550	2-(3,4-Dimethoxybenzyl)-7-{(1R)-1-[(1R)-1-hydroxyethyl]-4-phenylbutyl}-5-methyllimidazo[5,1-f][1,2,4]triazin-4(3H)-one	10011135	Cayman Chemicals
CNP		4019911	Bachem
Epinephrine	(R)-4-(1-hydroxy-2-(methylamino)ethyl)benzene-1,2-diol	E-4375	Sigma-Aldrich
Fluo-4 AM	N-[4-[6-[(acetyloxy)methoxy]-2,7-difluoro-3-oxo-3H-xanthen-9-yl]-2-[2-[2-[bis[2-[(acetyloxy)methoxy]-2-oxoethyl]amino]-5-methylphenoxy]ethoxy]phenyl]-N-[2-[(acetyloxy)methoxy]-2-oxoethyl]-glycine, (acetyloxy)methyl ester	F14201	Invitrogen
Fura-2 AM	2-[6-[bis[2-[(acetyloxy)methoxy]-2-oxoethyl]amino]-5-[2-[2-[bis[2-[(acetyloxy)methoxy]-2-oxoethyl]amino]-5-methylphenoxy]ethoxy]-2-benzofuranyl]-5-oxazolecarboxylic acid, (acetyloxy)methyl ester	F1201	Invitrogen

Isoprenaline	1-(3,4-Dihydroxyphenyl)-2-(isopropylamino)ethanol	I5627	Sigma-Aldrich
Norepinephrine	4-[(1R)-2-amino-1-hydroxyethyl]benzene-1,2-diol	A0937	Sigma-Aldrich
Streptozotocin	1-methyl-1-nitroso-3-[(2R,4R,5S,6R)-2,4,5-trihydroxy-6-(hydroxymethyl)oxan-3-yl]urea	S0130	Sigma-Aldrich

Table S5: Resources of cell culture materials for human induced pluripotent stem cell-derived cardiomyocytes.

**Substance**	**Catalogue no.**	**Supplier**
L-ascorbic acid 2-phosphate	A8960-5G	Sigma-Aldrich
B27 supplement	17504044	Thermo Fischer Scientific
CHIR99021	MY-10182	MedChemExpress
collagenase B	LS004147	Worthington Biochemical
E8 medium	A1517001	Thermo Fischer Scientific
FCS	F7524	Sigma-Aldrich
Geltrex	A1413302	Thermo Fischer Scientific
human recombinant albumin	A9731-10G	Sigma-Aldrich
IWP2	MY-13912	MedChemExpress
D-(+)-glucose supplement	G8769-100ML	Sigma-Aldrich
RPMI-Medium 1640	72400021	Thermo Fischer Scientific
Thiazovivin	MY-13257	MedChemExpress
Trypsin/EDTA	25200056	Thermo Fischer Scientific
Versene	15040033	Thermo Fischer Scientific

Supplementary Data D1

Band intensity and fold change of the housekeeping protein GAPDH in human patients with dilated cardiomyopathy and insulin-requiring diabetes (DiabDCM), non-diabetic patients with dilated cardiomyopathy (DCM) and non-failing (NF) hearts.

Representative human Western blots

NF
DiabDCM
DCM

samples 1-3 of 6

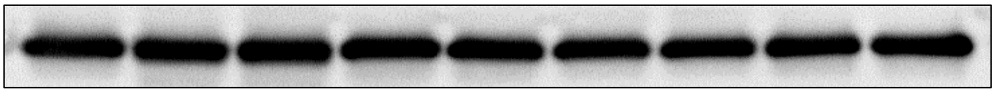

sample ID	II/6	III/26	I/6	627	977	989	2476	2622	2809
band intensity	19933327	21626541	21625773	22305068	21976692	20507716	20851287	20124923	25582627
mean NF	21061880.3								
fold change	0.9464	1.0268	1.0268	1.0590	1.0434	0.9737	0.9900	0.9555	1.2146

---

samples 4-6 of 6

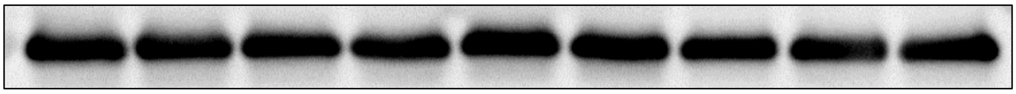

sample ID	IV/23	IV/25	V/9	2179	2362	2417	2852	2873	2575
band intensity	24774438	21972715	25207551	20987512	27450453	24393570	23711935	19246400	24479402
mean NF	23984901.3								
fold change	1.0329	0.9161	1.0510	0.8750	1.1445	1.0170	0.9886	0.8024	1.0206

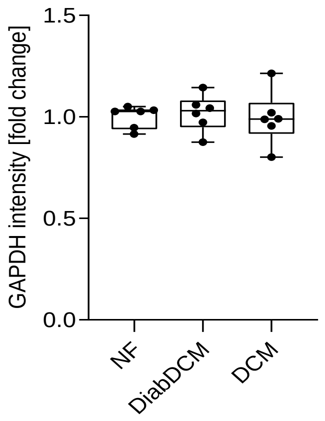

Band intensity and fold change of the housekeeping protein GAPDH in failing hearts from STZ-treated mice (STZ) or vehicle-injected mice (Con).

Representative mouse Western blots

Con
STZ

samples 1-5 of 8 or 10

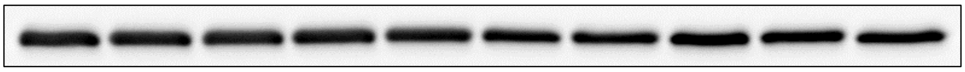

sample ID	6457	6458	6333	6273	6278	7890	7892	8018	7891	8358
band intensity	37647116	37219117	35907947	38508384	38012803	33778287	37316521	45605378	41963384	41750495
mean Con	37459073.4									
fold change	1.0050	0.9936	0.9586	1.0280	1.0148	0.9017	0.9962	1.2175	1.1202	1.1146

samples 6-8 or 6-10 of 8 or 10

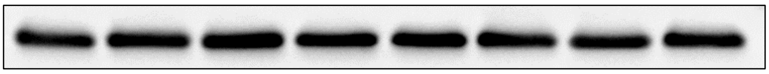

sample ID	7039	7137	6969	8304	7869	8305	8698	8357
band intensity	21287018	25030023	31150467	27852934	28036973	23363448	21705339	23481332
mean Con	25822502.7							
fold change	0.8244	0.9693	1.2063	1.0786	1.0858	0.9048	0.8406	0.9093

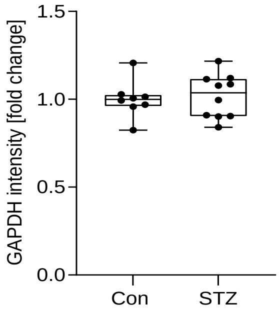

Supplemental Methods

Animal studies

The animal experiments comply with the ARRIVE 2.0 guidelines and Directive 2010/63/EU of the European Parliament on the protection of animals used for scientific purposes and were approved by the Animal Welfare Committee of TU Dresden and the Landesdirektion Dresden, Germany (TV vG 8/2022, TVV 60/2022, TVV 49/2020). The mice were housed in groups of 3-5 in standard cages at the Experimental Center of the Faculty of Medicine, TU Dresden under controlled temperature and humidity with a 12-h light/dark cycle, under specific pathogen-free conditions, and with free access to water and the food.

Genetically modified mice

Mice with a cardiomyocyte-specific deletion of PDE2 (KO) were generated using the Cre/loxP system in a C57BL/6N background, as previously published[3]. α-MHC Cre was used to ensure cardiac specificity.

Randomisation and blinding procedures

Sample size calculation: The sample size was calculated based on a priori analysis by GPower 3.1.9.2 software (two-tailed, with a statistical power of 80% and $\alpha = 0.05$; the effect size was determined based on previous experimental results [3] and a review of the literature).

Randomisation/Blinding: The mice were labelled using the animal facility labelling system and randomly assigned to various experimental groups using an online, software-based random number generator (https://www.randomizer.org/). Mice of control and experimental groups used in the study were littermates. A single experienced operator carried out the mouse cardiomyocyte isolation procedure using a blinded approach. The

operator, who was blinded to the genotype, randomly assigned the cells to the treatment groups. The order in which the treated cells were measured was randomised each day to minimise confounders. Throughout the data analysis process, the analysts were blinded to the genotypes and the experimental group assignments.

Inclusion criteria:

- Echocardiography: absence of signs of impairments or abnormal behaviour
- *ex vivo* ECG recordings: mice without signs of impairments or abnormal behaviour
- Isolation of single cardiomyocytes (for Ca^{2+} imaging, patch-clamp and cellular contractility studies): mice without signs of impairments or abnormal behaviour

Exclusion criteria:

- Echocardiography: no exclusion criteria applied
- *ex vivo* ECG recordings: hearts without ECG stabilization after 15 min
- Isolation of single cardiomyocytes (for Ca^{2+} imaging, patch-clamp and cellular contractility studies): none

No animals, cells or data points were excluded in the current study unless there was clear evidence of technical failure during the experiments.

Diabetes induction

Diabetes was induced as previously described[5, 9]. In brief, the mice received a daily i.p. injection of 50 µg/g streptozotocin (STZ, Sigma-Aldrich) dissolved in a 50 mM sodium citrate buffer (pH = 4.5), once-daily for 5 consecutive days, or vehicle only (control group). The mice were randomly assigned to the groups beforehand. The STZ injections were followed by a 30-d waiting period to allow diabetes to develop. Blood glucose and body weight were measured daily during the STZ injection phase and subsequently twice weekly. When blood glucose levels exceeded 500 mg/dl, 1 IU human basal insulin

(Huminsulin normal 100, Eli Lilly and Company) dissolved in 50 µl of water was injected subcutaneously. Those responsible for scoring or assessment were blinded to genotypes.

Blood glucose and HbA1c quantification

One drop of tail venous blood was collected for blood glucose measurement using the Accu-Chek Aviva blood glucose monitoring system (Roche). Following anaesthesia with 0.2 ml sodium thiopental 5% (w/v) and subsequent heart excision, 100 µl of abdominal whole blood were collected and mixed with 1.1 µl of 0.5 M EDTA solution (pH 9.0, Invitrogen). The samples were frozen at -20 °C until further use. Glycated haemoglobin A1c (HbA1c) levels were quantified by ELISA using a mouse HbA1c assay kit (catalog no. 80310, Crystal Chem) according to the manufacturer's instructions. Briefly, the whole blood samples were lysed and then subjected to protease digestion to release glycated valines from the haemoglobin beta chains. The glycated N-terminal valines were then specifically cleaved by the enzyme fructosyl valine oxidase to generate hydrogen peroxide, which was quantified using a horseradish peroxidase-catalysed reaction and a chromogen. Optical density (OD) at 700 nm was measured using a Synergy HTX (BioTek Instruments), and HbA1c concentrations were determined using a calibration curve.

Echocardiography

Echocardiography was performed as previously described [12]. The animals were anaesthetised with 2% (v/v) isoflurane and continuously monitored. A surface ECG was obtained using limb electrodes, and body temperature (37 °C warming plate, anal probe) was closely monitored during the procedure. Ultrasound images were acquired in the supine position using a Vevo 3100 imaging system (VisualSonics). A 2D echocardiographic study was performed using both the parasternal long-axis and short-axis views at the mid-papillary muscle and apical levels to assess diastolic and systolic function. Fractional area shortening (FAS, %) was calculated from left ventricular

endocardial areas in end-diastole and end-systole. The left ventricular ejection fraction (LVEF, %) was calculated using the left ventricular length at end-diastole and end-systole, as well as the left ventricular areas at end-diastole and end-systole. All echocardiographic images were analysed by an experienced operator who was blinded to the experimental group assignment.

ECG measurements of *ex vivo* Langendorff perfused hearts

The procedure was performed, with some modifications to isolated murine hearts, as described previously[10]. Mice (13–16 weeks old, sex-matched) were anaesthetised with 0.2 ml of 5% (w/v) sodium thiopental and their hearts were rapidly excised and placed in ice-cold, Ca^{2+}-free PBS. Following cannulation of the ascending aorta, the hearts were perfused with ice-cold, Ca^{2+}-free PBS to remove blood. Then, a silk 6-0 suture was loosely threaded around the left anterior descending coronary artery (LAD), 3-4 mm distal from the left atrial appendage. The cannulated hearts were then perfused retrogradely by gravity flow on a Langendorff perfusion system at 37 °C with a Krebs-Henseleit buffer solution containing the following components (in mM): NaCl: 118.5, $NaHCO_3$: 25, KCl: 3, $CaCl_2$: 2.4, $MgSO_4$: 1.2. NaH_2PO_4: 1.21, glucose: 11.1, with physiological catecholamine concentrations (10 nM norepinephrine; 3.5 nM epinephrine)[6]. Additionally, the hearts were perfused with vosoritide (VO, 200 nM) (Hycultec) or VO plus BAY 60-7550 (BAY, 300 nM) (Cayman Chemical). After stabilisation for 15 min, ischaemia was induced by ligating the left anterior descending coronary artery (LAD) with a PE10 tube inserted into a transient suture, which was then tightened to compress the LAD and stop the flow of blood. After 30 min, the tube was removed to allow reperfusion for a further 30 min. A bipolar electrocardiogram was recorded at 1 kHz using a bio amplifier (BioAmp, ADInstruments) connected to a PowerLab system (ADInstruments) and a PC with LabChart (ADInstruments). The positive electrode was connected close to the right atrial

appendage and the negative electrode connected to the left ventricle. QT intervals were analysed from the ECGs prior to occlusion by averaging 3 randomly selected QT distances from the recordings. The ECG data were analysed by reviewers who were blinded to treatment and genotype.

Isolation of adult mouse cardiomyocytes

Ventricular myocytes were obtained from 13–16 weeks old, sex-matched mice as previously described [2]. Animals were anaesthetised by i.p. injection of 0.2 ml of 5% (w/v) sodium thiopental, and the heart was rapidly excised and placed in ice-cold, Ca^{2+}-free PBS. The ascending aorta was cannulated, and the heart was perfused for 2 min with perfusion buffer containing the following components (in mM): NaCl: 113, KCl: 4.7, $MgSO_4$: 1.2, KH_2PO4: 0.6, NaH_2PO_4: 0.6, $NaHCO_3$: 12, $KHCO_3$: 10, HEPES: 10, glucose: 5.5, BDM: 10, taurine: 30, phenol red: 0.0425, pH adjusted to 7.4 with KOH. For enzymatic dissociation, the heart was perfused with perfusion buffer containing 0.04 mg/ml Liberase^{TM}, research grade (Roche Diagnostics), 0.025% trypsin and 12.5 µM $CaCl_2$ for 12.5 min at 37°C. Once flaccid, the ventricular and atrial myocardium were separated. The ventricular myocardium was cut into small pieces and dispersed in the perfusion buffer. The digestion was terminated by addition of BSA, and the Ca^{2+} concentration was gradually increased from 32 µM to 960 µM, in 4-min intervals. For further experiments, the cardiomyocytes were plated on laminin-coated coverslips (2.5 cm) for 1 h or centrifuged at 4000 xg, room temperature for 3 min and snap-frozen immediately.

Measurement of SR Ca^{2+} sparks

Ca^{2+} spark (CaSp) measurement was performed as previously described [3, 11]. Ventricular myocytes were plated on laminin-coated coverslips and allowed to settle for 1 h at 37 °C, followed by a 20-min incubation at room temperature with 5 µM Fluo-4 AM (catalogue no. F14201, Invitrogen) dissolved in 2 mM Ca^{2+} Tyrode, containing (in mM):

NaCl: 140, KCl: 4, $MgCl_2$: 1, HEPES: 10, glucose: 10, $CaCl_2$: 2, NaH_2PO_4: 0.33 (pH adjusted to 7.4 with NaOH). To remove excess Fluo-4 AM and allow adequate de-esterification, cardiomyocytes were placed in Tyrode solution containing the respective experimental compounds: isoprenaline (Iso, 10 nM, or 30 nM), Iso plus CNP (1 µM), Iso plus vosoritide (VO, 1 µM), Iso plus CNP plus BAY 60-7550 (BAY, 100 nM), or Iso plus VO plus BAY for 7 min. CaSp measurements were carried out on a laser scanning confocal microscope (LSM 880 Pascal, Zeiss) with a 63x oil-immersion objective. Fluo-4 was excited by an argon ion laser (488 nm). Emitted fluorescence was collected through a 505 nm long-pass emission filter. Fluorescence images were recorded in the line-scan mode (scan line width: 45 µm, 512 pixels per line, pixel dwell time: 0.64 µs, 40,000 unidirectional line scans). Confocal line scans were performed at rest after a brief period of field stimulation to load the SR (1 Hz, 10 V for 10 sec). Experiments were performed at room temperature. CaSp were quantified in a standardised area comprising 22,000 scanning cycles starting 1 s after the last transient using SparkMaster plugin for ImageJ (NIH) and the following criteria: amplitude F/F0 ≥ 0.45, full width half maximum ≥ 0.79 µm and full duration half maximum ≥ 6.5 ms. Mean CaSp frequency (CaSpF) was calculated per 100 $\mu m^{-1} \cdot s^{-1}$. Mean Ca^{2+} leak per myocyte was calculated by multiplying CaSpF (per 100 $\mu m^{-1} \cdot s^{-1}$) with mean spark amplitude (F/F0), mean spark width (µm) and mean spark duration (ms). CaSp measurements in hiPSC-CMs were performed as previously described [3, 4, 8]. Briefly, the culture medium of hiPSC-CMs seeded on 22 mm coverslips was replaced by 5 µM Fluo-4 in 1.8 mM Ca^{2+} Tyrode solution containing (in mM): NaCl: 140, KCl: 5.4, $CaCl_2$: 1.8, $MgCl_2$: 2.1, HEPES: 10, glucose: 10 (pH adjusted to 7.3). Cells were washed twice with Tyrode solution and incubated for 10 min in Tyrode solution alone, or Tyrode solution containing isoprenaline (Iso, 100 nM), Iso plus vosoritide (VO, 1 µM), or Iso plus VO plus BAY 60-7550 (BAY). Recording of CaSp was performed using an LSM 880 confocal microscope in line-scan mode (scan line width: 45

μm, 512 pixels per line, pixel dwell time: 0.64 μs, 20,000 cycles). During measurement, hiPSC-CMs were paced at 0.5 Hz, 10 mV, 10 ms pulse duration. CaSp were quantified in standardised areas between induced Ca^{2+} transients using SparkMaster plugin of ImageJ (NIH) and using the same criteria as for CaSp detected in murine cardiomyocytes.

Patch-clamp experiments

Patch-clamp experiments were performed as previously described [3]. The ion current was measured by whole-cell patch-clamp technique using an EPC9 or EPC10 amplifier (HEKA) connected to a PC and controlled via Patchmaster software (HEKA). Pipette resistance was 2-3 MΩ when filled with the respective intracellular solution. Membrane capacitance (C_m) and series resistance (R_s) were determined using the automatic routines of the Patchmaster software before C_m compensation. R_s was in the range of ~5 MΩ, was not permitted to exceed 12 MΩ and was compensated by 85%. The reference electrode was placed in pipette solution within a separate chamber connected to the bath solution via an agar bridge filled with pipette solution. Pipette potential (V_{Pip}) and V_m were corrected for liquid junction potentials at the bridge-bath junction. All experiments were performed at room temperature and currents were normalized to cell capacitance. Patch clamp data were analysed using the Fitmaster software (HEKA) and Excel (Microsoft).

L-Type Ca^{2+} current ($I_{Ca,L}$): Pipettes were filled with an intracellular solution containing (in mM): CsCl 110, EGTA 10, $MgCl_2$ 5, TEA-Cl 20, HEPES 10 and Na_2-ATP 2 (pH adjusted to 7.2 with CsOH). Extracellular Tyrode solution contained (in mM): NaCl: 138, $CaCl_2$: 2, $MgCl_2$: 1, NaH_2PO_4: 0.33, glucose: 10, HEPES: 10 and KCl: 0 or 4 (pH adjusted to 7.3 with NaOH). To assess $I_{Ca,L}$ upon β-AR stimulation, cells were pre-incubated with bath solution containing either isoprenaline (Iso, 30 nM), Iso plus vosoritide (VO, 10 μM) or Iso plus VO plus BAY 60-7550 (BAY, 300 nM) ~10 min prior to every recording as indicated. Cells were held at -90 mV. I_{CaL} was elicitied by depolarising the cells every 3 s

from -60 to 70 mV in 10 mV increments for 600 ms, following a pre-pulse to -40 mV of 70 ms duration to inactivate voltage-dependent Na^+ currents. Cells were perfused with K^+-free Tyrode solution for 20 s prior to $I_{Ca,L}$ measurement to prevent inward rectifying K^+ currents. Maximal $I_{Ca,L}$ was recorded at 0 mV. $I_{Ca,L}$ was measured at the difference between peak inward current and the current at the end of the voltage pulse. Whole-cell currents were low-pass filtered at 1 kHz and sampled at 5 kHz.

Cellular action potentials: Pipettes were filled with an intracellular solution containing (in mM): glutamic acid 130, KCl 10, $MgCl_2$ 4, HEPES 10, Na_2ATP 2 (pH adjusted to 7.2 with KOH). Extracellular Tyrode solution contained (in mM): NaCl 138, KCl 4, $CaCl_2$ 2, $MgCl_2$ 1, NaH_2PO_4 0.33, glucose 10 and HEPES 10 (pH adjusted to 7.3 with NaOH). Cells were incubated for 10 min in bath solution containing either 10 µM vosoritide (VO) or not. To assess action potential properties, APs were evoked by brief current pulses (5 or 10 ms, 200-1000 pA) at 1 Hz.

Calcium imaging

Measurement of Ca^{2+} transients was performed as previously described [1, 3]. Cardiomyocytes were loaded with 5 µM Fura-2 AM dissolved in 2 mM Ca^{2+} Tyrode solution (for components see "Measurement of SR Ca^{2+} sparks") for 25 min at 37 °C. After washing, cardiomyocytes were incubated in 2 mM Ca^{2+} Tyrode containing isoprenaline (Iso, 30 nM), Iso plus vosoritide (VO, 10 µM), or Iso plus VO plus BAY 60-7550 (BAY, 300 nM) for an additional 5 min. The cells were then paced at 1 Hz for 30 s, followed by 2 Hz pacing for another 30 s to load the sarcoplasmic reticulum with Ca^{2+}. The Fura-2 ratio of Ca^{2+} transients was measured at 340 and 380 nm using an IonOptix system as described previously[3]. This was followed by replacement of the 2 mM Ca^{2+} Tyrode solution by Iso/ Iso+VO/ Iso+VO+BAY-supplemented $0Na^+/0Ca^{2+}$ Tyrode solution containing Li^+ and the RyR2 inhibitor tetracaine (1 mM, 30 s) to exclude trans-

sarcolemmal and intracellular SR Ca^{2+} fluxes. Spontaneous Ca^{2+} waves (SCW) were measured and quantified during tetracaine washout for 30 s with $0Na^{+}/0Ca^{2+}$ Tyrode solution. Caffeine (10 mM) was then applied to induce complete release of SR Ca^{2+}. Analysis was performed using the IonWizard (IonOptix), with Fura-2 ratios filtered (Butterworth Lowpass, Cutoff frequency: 100.000, Number of Poles: 2) following background signal subtraction. Ca^{2+} transient amplitudes were measured as the difference between peak systolic and end diastolic ratios. Transient properties were determined upon assessing transient peak velocity and transient decay.

***Ex vivo* perfusion for Western blot**

13–16-week-old, sex-matched mice were randomised and anaesthetised with 0.2 ml of 5% (w/v) sodium thiopental. The hearts were rapidly excised and placed into ice-cold, Ca^{2+}-free PBS. Following cannulation of the ascending aorta, the hearts were perfused with ice-cold, Ca^{2+}-free PBS to remove blood and a silk 6-0 suture was loosely threaded around the left anterior descending coronary artery (LAD), 3-4 mm distal from the left atrial appendage. The cannulated hearts were then retrogradely perfused by gravity flow on a Langendorff perfusion system for 1:15 h at 37 °C with a Krebs-Henseleit buffer solution (for components see “ECG measurements of *ex vivo* Langendorff perfused hearts”) supplemented with catecholamines (10 nM norepinephrine, 3.5 nM epinephrine). Additionally, the hearts were perfused with the same buffer containing either vosoritide (VO, 200 nM) or VO (200 nM) plus BAY 60-7550 (BAY, 300 nM). After perfusion, the heart was dissected, and the tissue was snap-frozen immediately.

Protein isolation and immunoblot

Isolated cell pellets or frozen tissue samples were homogenised in RIPA lysis buffer containing 150 mM NaCl, 50 mM Tris-HCl, 1 mM EDTA, 1% IGEPAL CA-630, 0.25% sodium deoxycholate, 0.1% SDS as well as phosphatase and protease inhibitors

(PhosSTOP^{TM}, catalogue no. 04906837001, cOmplete^{TM} Tablets, Mini, catalogue no. 04693124001, Roche) using mechanical disruption (QIAGEN TissueLyser LT) for 4 min at 50 Hz. After centrifugation (4 °C, 5.000 xg, 15 min), the protein-containing supernatant was collected. Protein concentrations were measured using the Pierce^{TM} BCA Protein Assay Kit (catalogue no. 23225, Thermo Scientific) according to the manufacturer's instructions and a Synergy HTX microplate reader (BioTek Instruments). For immunoblotting, total protein extracts (50 µg for RyR2 and pRyR2 (Ser2814), 20 µg for other proteins) were run on a 6% SDS-PAGE (RyR2, pRyR2 (Ser2814)) or tris-tricine gel (other proteins) and transferred onto a nitrocellulose membrane. Successful transfer was confirmed by PonceauS staining. After blocking the membranes with 5% BSA (for phosphorylation sites), or 5% milk buffer (for total proteins) in 0.1% TBS-T for 1 h at room temperature, membranes were incubated with primary antibodies (for dilutions see table S3) overnight at 4 °C. On the following day, after washing, the membranes were probed with secondary HRP-coupled antibodies (see table S3) for 1 h at room temperature. After the final washing step, membranes were incubated with ECL substrate (SuperSignal^{TM} West Femto Maximum Sensitivity Substrate, catalogue no. 34094, resp. SuperSignal^{TM} West Dura Extended Duration Substrate, catalogue no. 37071, Thermo Scientific) and images were acquired using a Fusion FX chemiluminescence imaging system (Vilber). Densitometric analysis was performed using FusionCapt Advance software (Vilber), and relative protein expression were calculated as fold change relative to the control group.

cAMP quantification by ELISA

cAMP ELISA was performed as previously published[3]. Freshly isolated ventricular cardiomyocytes were treated with 2 mM Ca^{2+} Tyrode solution (for components see "Measurement of SR Ca^{2+} sparks") supplemented with isoprenaline (10 nM), vosoritide (1 µM) or BAY 60-7550 (100 nM) for 8 min at 37 °C. Cells were collected and centrifuged

(900 g, 2 min, 4 °C) and immediately lysed in 300 µl 0.1 M HCl (15 min) to stop endogenous phosphodiesterase activity and stabilise the released cAMP. Cell debris was removed upon centrifugation (600 g, 10 min at 4 °C). cAMP content in the supernatant was determined using the Direct cAMP ELISA Kit (RRID: AB_2890930, Enzo Life Sciences) according to the manufacturer's instructions. Samples and standards were acetylated before performing the ELISA assay using acetic anhydride and trimethylamine. Upon termination of the colorimetric reaction, absorbance was detected at 405 nm using a Synergy HTX microplate reader (BioTek Instruments). A standard curve was constructed by plotting absorbance against the respective logarithmic cAMP concentrations to calculate sample cAMP concentrations. Samples were measured in duplicates and averaged. Sample cAMP content was normalized to protein content and presented as pmol/mg protein, following determination of protein concentrations by BCA assay using the Pierce™ BCA Protein Assay Kit (catalogue no. 23225, Thermo Scientific).

Cellular contractile function

Cellular contractile function was measured as previously described [3]. Cardiomyocytes were pre-incubated with 2 mM Ca^{2+} Tyrode solution (for components see "Measurement of SR Ca^{2+} sparks") supplemented with respective experimental compounds: vosoritide (VO, 1 µM), isoprenaline (Iso, 10 nM), or Iso plus VO for 5 min at 37 °C on laminin-coated glass coverslips. Rod-shaped cardiomyocytes displaying clear sarcomere banding were selected for measurement of mechanical properties using a SarcLen Myocam system (IonOptix). During field stimulation (1 Hz, 10 V, 10 ms, 37 °C), the cells were continuously perfused with the corresponding VO/Iso/Iso+VO-supplemented Tyrode solution. Using IonWizard software, sarcomere shortening was calculated as the ratio of the twitch amplitude to the end-diastolic sarcomere length. Contractile kinetics were quantified by

measuring contraction velocity, and the diastolic function was assessed by evaluating relaxation velocity.

Differentiation and cultivation of human induced pluripotent stem cell-derived cardiomyocytes (hiPSC-CMs)

The human iPSC line iBM76.3 (UMGi005-A, clone 3) used in this study was reprogrammed from mesenchymal stem cells of a healthy donor using STEMCCA lentiviral system and has been characterised previously[4]. The generation of human iPSC lines was approved by the Ethics Committees of the University Medical Center in Göttingen (approval numbers 21/1/11 and 10/9/15) and TU Dresden (EK422092019) and was performed in accordance with the approved guidelines.

Human iPSCs were cultured in E8 medium (Thermo Fischer Scientific) on Geltrex-coated (Thermo Fischer Scientific) cell culture plates at 37 °C in an atmosphere containing 5% CO_2. Culture medium was replaced daily. At 80-90% confluency, cells were passaged using Versene (Thermo Fischer Scientific). Differentiation of hiPSCs into hiPSC-derived cardiomyocytes (hiPSC-CMs) was performed as previously described [7]. In brief, hiPSCs were seeded into 12-well plates and grown to confluency of 80-90%. Cardiac differentiation was carried out using cardio differentiation medium, consisting of RPMI 1640 with Glutamax and HEPES (Thermo Fischer Scientific) supplemented with 0.2 mg/ml L-ascorbic acid 2-phosphate (Sigma-Aldrich) and 0.5 mg/ml human recombinant albumin (Sigma-Aldrich). To initiate differentiation (day 0), E8 medium was replaced with cardio differentiation medium containing 4 µM CHIR99021 (a GSK3β inhibitor, Merck Millipore) for 48 h. Thereafter, medium was changed to cardio differentiation medium supplemented with 5 µM IWP2 (a WNT signaling inhibitor, Merck Millipore) for further 48 h, followed by culture in cardio differentiation medium for 4 d with medium change after 48 h. From day 8, cells were cultured in RPMI 1640 with Glutamax and HEPES

containing 2% B27 supplement (RPMI/B27 medium, Thermo Fischer Scientific) with medium changes every other day. First spontaneous contractions were observed on days 8–9. Between days 14–16, hiPSC-CMs were replated into Geltrex-coated 6-well plates. Therefore, hiPSC-CMs were incubated in RPMI 1640 with Glutamax and HEPES containing 1 mg/ml collagenase B (Worthington Biochemical) for 1 hour at 37°C. Detached hiPSC-CM layers were collected and dissociated in 0.25% trypsin/EDTA (Thermo Fischer Scientific) for 8 minutes at 37°C. Cells were resuspended in cardio digestion medium, composed of RPMI/B27 medium with 20% FCS (Sigma-Aldrich) and 2 µM thiazovivin (Millipore). Subsequently, hiPSC-CMs were centrifuged at 200 x g for 5 min, resuspended in cardio digestion medium, and seeded into Geltrex-coated 6-well plates at a density of 700,000 cells per well. On the following day, medium was replaced by RPMI/B27 medium and exchanged every other day. Cells were cultured until day 49.

For CaSp measurement, hiPSC-CMs were replated on Geltrex-coated 22 mm glass coverslips in 6-well plates at a density of 200,000 cells per well and cultured in RPMI/B27 medium for at least 1 week to allow recovery. Thereafter, cells were cultured in RPMI/B27 medium with standard (11 mM glucose, NG) or high glucose (22 mM glucose, HG) concentrations for 7 days with medium changes every other day.

Multi-electrode array experiments

All multi-electrode array (MEA) recordings were conducted using a Maestro Edge system and AxIS Navigator software (Axion BioSystems) at 37°C in an atmosphere containing 5% CO_2, with a sampling rate of 12,500 Hz [7]. Cells were resuspended cardio digestion medium, and 25,000 cells were seeded onto the electrode area of Geltrex-coated CytoView 24-well MEA plates (Axion BioSystems). After 60 minutes, 1 ml of cardio-digestion medium was gently added to each well. The iPS-CMs were cultured for 4 days in B27 medium, followed by 7 days in RPMI/B27 medium containing 22 mM glucose (HG

medium), with medium change every 2 days. Cells were then treated with vosoritide (VO, 1 µM), isoprenaline (Iso, 100 nM) or Iso plus VO diluted in HG medium, and recordings were performed after 10 min of incubation at 37°C in 5% CO_2.

References

1. Bassani RA, Bers DM (1995) Rate of diastolic Ca release from the sarcoplasmic reticulum of intact rabbit and rat ventricular myocytes. Biophys J 68:2015-2022 doi:10.1016/s0006-3495(95)80378-4
2. Börner S, Schwede F, Schlipp A, Berisha F, Calebiro D, Lohse MJ, Nikolaev VO (2011) FRET measurements of intracellular cAMP concentrations and cAMP analog permeability in intact cells. Nature protocols 6:427-438 doi:10.1038/nprot.2010.198
3. Cachorro E, Günscht M, Schubert M, Sadek MS, Siegert J, Dutt F, Bauermeister C, Quickert S, Berning H, Nowakowski F, Lämmle S, Firneburg R, Luo X, Künzel SR, Klapproth E, Mirtschink P, Mayr M, Dewenter M, Vettel C, Heijman J, Lorenz K, Guan K, El-Armouche A, Wagner M, Kämmerer S (2023) CNP Promotes Antiarrhythmic Effects via Phosphodiesterase 2. Circulation research 132:400-414 doi:10.1161/circresaha.122.322031
4. Cyganek L, Tiburcy M, Sekeres K, Gerstenberg K, Bohnenberger H, Lenz C, Henze S, Stauske M, Salinas G, Zimmermann WH, Hasenfuss G, Guan K (2018) Deep phenotyping of human induced pluripotent stem cell-derived atrial and ventricular cardiomyocytes. JCI Insight 3 doi:10.1172/jci.insight.99941
5. Graniel-Amador MA, Torres-Rodriguez HF, Martinez-Mendoza RE, Vargas-Munoz VM, Acosta-Gonzalez RI, Castaneda-Corral G, Munoz-Islas E, Jimenez-Andrade JM (2022) Effect of chronic lithium on mechanical sensitivity and trabecular bone loss induced by type-1 diabetes mellitus in mice. Biometals 35:1033-1042 doi:10.1007/s10534-022-00421-5
6. Grouzmann E, Cavadas C, Grand D, Moratel M, Aubert JF, Brunner HR, Mazzolai L (2003) Blood sampling methodology is crucial for precise measurement of plasma catecholamines concentrations in mice. Pflugers Archiv : European journal of physiology 447:254-258 doi:10.1007/s00424-003-1140-x
7. Li W, Luo X, Poetsch MS, Oertel R, Nichani K, Schneider M, Strano A, Hasse M, Steiner RP, Cyganek L, Hettwer K, Uhlig S, Simon K, Guan K, Schubert M (2022) Synergistic Adverse Effects of Azithromycin and Hydroxychloroquine on Human Cardiomyocytes at a Clinically Relevant Treatment Duration. Pharmaceuticals (Basel) 15 doi:10.3390/ph15020220
8. Luo X, Li W, Künzel K, Henze S, Cyganek L, Strano A, Poetsch MS, Schubert M, Guan K (2020) IP3R-Mediated Compensatory Mechanism for Calcium Handling in Human Induced Pluripotent Stem Cell-Derived Cardiomyocytes With Cardiac Ryanodine Receptor Deficiency. Frontiers in cell and developmental biology 8:772 doi:10.3389/fcell.2020.00772
9. Motyl KJ, McCauley LK, McCabe LR (2012) Amelioration of type I diabetes-induced osteoporosis by parathyroid hormone is associated with improved osteoblast survival. J Cell Physiol 227:1326-1334 doi:10.1002/jcp.22844
10. Stables CL, Curtis MJ (2009) Development and characterization of a mouse in vitro model of ischaemia-induced ventricular fibrillation. Cardiovascular research 83:397-404 doi:10.1093/cvr/cvp068

11. Trafford AW, Díaz ME, O'Neill SC, Eisner DA (1995) Comparison of subsarcolemmal and bulk calcium concentration during spontaneous calcium release in rat ventricular myocytes. The Journal of physiology 488 (Pt 3):577-586 doi:10.1113/jphysiol.1995.sp020991
12. Vettel C, Lindner M, Dewenter M, Lorenz K, Schanbacher C, Riedel M, Lammle S, Meinecke S, Mason FE, Sossalla S, Geerts A, Hoffmann M, Wunder F, Brunner FJ, Wieland T, Mehel H, Karam S, Lechene P, Leroy J, Vandecasteele G, Wagner M, Fischmeister R, El-Armouche A (2017) Phosphodiesterase 2 Protects Against Catecholamine-Induced Arrhythmia and Preserves Contractile Function After Myocardial Infarction. Circulation research 120:120-132 doi:10.1161/circresaha.116.310069

NOTE: Please save this file locally before filling in the table, DO NOT work on the file within your internet browser as changes will not be saved. Adobe Acrobat Reader (available free here) is recommended for completion.

The ARRIVE guidelines 2.0: author checklist

The ARRIVE Essential 10

These items are the basic minimum to include in a manuscript. Without this information, readers and reviewers cannot assess the reliability of the findings.

Item		Recommendation	Section/line number, or reason for not reporting
**Study design**	1	For each experiment, provide brief details of study design including:	
		a. The groups being compared, including control groups. If no control group has been used, the rationale should be stated.	Online materials and corresponding figure legends
		b. The experimental unit (e.g. a single animal, litter, or cage of animals).	Corresponding figure legends
**Sample size**	2	a. Specify the exact number of experimental units allocated to each group, and the total number in each experiment. Also indicate the total number of animals used.	Corresponding figure legends
		b. Explain how the sample size was decided. Provide details of any *a priori* sample size calculation, if done.	Online materials page 15
**Inclusion and exclusion criteria**	3	a. Describe any criteria used for including and excluding animals (or experimental units) during the experiment, and data points during the analysis. Specify if these criteria were established *a priori*. If no criteria were set, state this explicitly.	Online material page 15
		b. For each experimental group, report any animals, experimental units or data points not included in the analysis and explain why. If there were no exclusions, state so.	Online material page 15
		c. For each analysis, report the exact value of *n* in each experimental group.	Corresponding figure legends
**Randomisation**	4	a. State whether randomisation was used to allocate experimental units to control and treatment groups. If done, provide the method used to generate the randomisation sequence.	Online material page 15
		b. Describe the strategy used to minimise potential confounders such as the order of treatments and measurements, or animal/cage location. If confounders were not controlled, state this explicitly.	Online materials page 11
**Blinding**	5	Describe who was aware of the group allocation at the different stages of the experiment (during the allocation, the conduct of the experiment, the outcome assessment, and the data analysis).	Online material page 15
**Outcome measures**	6	a. Clearly define all outcome measures assessed (e.g. cell death, molecular markers, or behavioural changes).	Online materials and Methods
		b. For hypothesis-testing studies, specify the primary outcome measure, i.e. the outcome measure that was used to determine the sample size.	Online materials and Methods
**Statistical methods**	7	a. Provide details of the statistical methods used for each analysis, including software used.	Methods and corresponding figure legends
		b. Describe any methods used to assess whether the data met the assumptions of the statistical approach, and what was done if the assumptions were not met.	Methods and corresponding figure legends
**Experimental animals**	8	a. Provide species-appropriate details of the animals used, including species, strain and substrain, sex, age or developmental stage, and, if relevant, weight.	Online materials page 9 (Table S2)
		b. Provide further relevant information on the provenance of animals, health/immune status, genetic modification status, genotype, and any previous procedures.	Online materials page 9
**Experimental procedures**	9	For each experimental group, including controls, describe the procedures in enough detail to allow others to replicate them, including:	
		a. What was done, how it was done and what was used.	Online Materials and Major resources table
		b. When and how often.	Online Materials and Major resources table
		c. Where (including detail of any acclimatisation periods).	Online Materials page 14
		d. Why (provide rationale for procedures).	Results and Online Materials
**Results**	10	For each experiment conducted, including independent replications, report:	
		a. Summary/descriptive statistics for each experimental group, with a measure of variability where applicable (e.g. mean and SD, or median and range).	Corresponding figure legends
		b. If applicable, the effect size with a confidence interval.	Corresponding figure legends
